# Harmonization and qualification of an IFN-γ Enzyme-Linked ImmunoSpot assay (ELISPOT) to measure influenza-specific cell-mediated immunity within the FLUCOP consortium

**DOI:** 10.3389/fimmu.2022.984642

**Published:** 2022-09-08

**Authors:** Gwenn Waerlop, Geert Leroux-Roels, Teresa Lambe, Duncan Bellamy, Donata Medaglini, Elena Pettini, Rebecca Jane Cox, Mai-Chi Trieu, Richard Davies, Geir Bredholt, Emanuele Montomoli, Elena Gianchecchi, Frédéric Clement

**Affiliations:** ^1^ Center for Vaccinology (CEVAC), University Hospital, Ghent University, Ghent, Belgium; ^2^ Nuffield Department of Medicine, The Jenner Institute, University of Oxford, Oxford, United Kingdom; ^3^ Laboratory of Molecular Microbiology and Biotechnology, Department of Medical Biotechnologies, University of Siena, Siena, Italy; ^4^ Influenza Centre, Department of Clinical Science, University of Bergen, Bergen, Norway; ^5^ Department of Molecular and Developmental Medicine, University of Siena, Siena, Italy; ^6^ VisMederi srl, Siena, Italy

**Keywords:** IFN-γ ELISpot, cell-mediated immunity, assay qualification, assay harmonization, influenza

## Abstract

Influenza continues to be the most important cause of viral respiratory disease, despite the availability of vaccines. Today’s evaluation of influenza vaccines mainly focuses on the quantitative and functional analyses of antibodies to the surface proteins haemagglutinin (HA) and neuraminidase (NA). However, there is an increasing interest in measuring cellular immune responses targeting not only mutation-prone surface HA and NA but also conserved internal proteins as these are less explored yet potential correlates of protection. To date, laboratories that monitor cellular immune responses use a variety of in-house procedures. This generates diverging results, complicates interlaboratory comparisons, and hampers influenza vaccine evaluation. The European FLUCOP project aims to develop and standardize assays for the assessment of influenza vaccine correlates of protection. This report describes the harmonization and qualification of the influenza-specific interferon-gamma (IFN-γ) Enzyme-Linked ImmunoSpot (ELISpot) assay. Initially, two pilot studies were conducted to identify sources of variability during sample analysis and spot enumeration in order to develop a harmonized Standard Operating Procedure (SOP). Subsequently, an assay qualification study was performed to investigate the linearity, intermediate precision (reproducibility), repeatability, specificity, Lower and Upper Limits of Quantification (LLOQ-ULOQ), Limit of Detection (LOD) and the stability of signal over time. We were able to demonstrate that the FLUCOP harmonized IFN-γ ELISpot assay procedure can accurately enumerate IFN-γ secreting cells in the analytical range of 34.4 Spot Forming Units (SFU) per million cells up to the technical limit of the used reader and in the linear range from 120 000 to 360 000 cells per well, in plates stored up to 6 weeks after development. This IFN-γ ELISpot procedure will hopefully become a useful and reliable tool to investigate influenza-specific cellular immune responses induced by natural infection or vaccination and can be an additional instrument in the search for novel correlates of protection.

## Introduction

Influenza continues to be the most important cause of viral respiratory disease associated with millions of hospitalizations and hundreds of thousands of deaths, despite the availability of vaccines (1, 2). The current seasonal human influenza vaccines are moderately effective in certain populations but require annual updating and administration. Additionally, the vaccine effectiveness varies depending on the match between the vaccine strains and the circulating strains. To overcome these shortcomings, there is an urgent need for new or improved influenza vaccines and efforts are already being made to design long-lasting universal influenza vaccines effective against different variants (3). Today’s evaluation of influenza vaccines mainly focuses on the quantitative and functional analyses of antibodies to haemagglutinin (HA) and neuraminidase (NA), the major surface glycoproteins of the virus. Cellular immune responses, primarily mediated by T cells, not only target the mutation-prone surface proteins but also internal proteins that are generally more conserved and shared by heterologous viral strains across influenza A subtypes. Consequently, vaccines inducing cellular immune responses are more likely to elicit broad protection against heterologous viral strains.

Reliable detection and quantification of these cellular responses are of key interest. Therefore, cellular immune assays need to be qualified and, if possible, even validated to demonstrate assay precision, robustness and specificity before being applied in clinical trials. To date, laboratories that monitor cellular immune responses use a variety of in-house procedures. This generates diverging results, complicates interlaboratory comparisons, and hampers influenza vaccine evaluation (4–6). The European FLUCOP project, supported by the Innovative Medicines Initiative Joint Undertaking (IMI-JU, under Grant Agreement 115672), aims to develop and standardize assays for the assessment of influenza vaccine correlates of protection (7). Within this consortium, efforts have been made to develop harmonized Standard Operating Procedures (SOPs) for influenza-specific interferon-gamma (IFN-γ) Enzyme-Linked Spot (ELISpot) and Intracellular Cytokine Staining (ICS) assays, followed by assay qualification. These two cell-mediated immunity (CMI) assays allow for the detection and quantification of antigen-specific cytokine responses to vaccination and infection. The ELISpot assay specifically aims at quantifying IFN-γ producing cells, such as CD4^+^ Th1 and CD8^+^ T cells which are the prime subsets of interest when examining influenza-specific responses induced by vaccination and/or infection. Note that other cell populations such as NK- and NK T cells can secrete IFN-γ and contribute to the spot formation in the plate ( (8, 9)). Depending on the research questions asked, other cytokines (e.g., IL-2, IL-4, IL-5) (10) can be investigated by ELISpot. The harmonization and qualification of the ICS assay are described in a separate report in this special topic issue (*Begue et al., 2022. Harmonization and Qualification of Intracellular Cytokine Staining to Measure Influenza-Specific CD4^+^ T Cell Immunity Within the FLUCOP Consortium (submitted)*).

This report describes the harmonization and qualification of the IFN-γ ELISpot assay. First, two pilot studies were conducted to identify sources of variability during sample analysis and spot enumeration with the aim of developing a harmonized SOP. Finally, an assay qualification study was performed to investigate the linearity, intermediate precision (reproducibility), repeatability, specificity, Lower and Upper Limits of Quantification (LLOQ-ULOQ), Limit of Detection (LOD) and the stability of signal over time.

## Materials and methods

### Samples

Peripheral blood mononuclear cells (PBMC) were isolated from 12 buffy coats obtained from healthy blood donors (Red Cross Flanders). PBMC were also isolated from blood sampled from 27 healthy volunteers that participated in a clinical vaccine trial that was carried out specifically for these studies. For this, venous blood was collected in heparin-coated blood collection tubes prior to and 7 days after the administration of a seasonal influenza vaccine (alfa-RIX-Tetra^©^ (season 2015/2016 or 2016/2017)). Ethical approvals for this study and the use of blood collected from Red Cross donors were given by the Ethical Committee of the Ghent University Hospital.

PBMC were isolated according to the standardized procedure *FLUCOP SOP for PBMC isolation and cryopreservation*, available as **Supplementary Material (Appendix 1)**. In brief, venous blood samples were diluted 1:2 in Hanks buffered salt solution (HBSS), and buffy coats were brought to a total volume of 300 mL in HBSS. PBMC were isolated by isopycnic centrifugation using Lymphoprep™. Subsequently, cells were washed twice in HBSS, suspended in freezing medium [10% dimethyl sulfoxide (DMSO)/90% fetal bovine serum (FBS)], frozen at a concentration of ≥ 5 to ≤ 20 million cells/mL within 24h (buffy coats) or 6h (whole venous blood samples) after blood collection and finally stored in liquid nitrogen until use.

All cryovials were identified with unique codes without any reference to their source. All samples later distributed to other laboratories were selected from this PBMC biobank based on their pre-examined CMI immune responses against influenza antigens.

### Pilot studies

#### Pilot study 1

In pilot study 1, different methods applied to analyse samples were carefully examined. A panel of 24 coded PBMC samples was distributed to 5 consortium partners. Each vial had a unique code allowing only the organizing center to link it with the original specimen identifier. Hereafter, sample identifiers 1 to 24 will be used. Participating laboratories were also provided with antigens for *in vitro* stimulations of the PBMC. These were recombinant Hemagglutinin (HA) H1 A/California/07/2009 (catalog number 3006) and recombinant Hemagglutinin (HA) B/Phuket/3073/2013 (catalog number 3006), both from Protein Sciences Corporation (Swiftwater, PA) and kindly provided by Sanofi. The recombinant proteins were produced in insect cells using the baculovirus expression vector system and purified to at least 90%. Each laboratory was asked to analyze the samples using their in-house procedure for IFN-γ ELISpot and preferred reagents. An online worksheet was filled out to collect information regarding the number of vials thawed, thawing medium, thawing medium temperature, thawing process, FBS validation status, cell counting technique, the technique applied for determination of cell viability, cell resting time and medium as well as the concentration of cells during the resting period (where applicable), culture medium, the use of self- or pre-coated plates, coating antibody, conjugated detection antibody, cell concentration in the well, stimulation/incubation time, substrate for staining, the process of washing the wells, stop reaction, ELISpot reader and related software, Quality Control (QC) process, validation criteria on background conditions, and any comments/deviations that may have occurred. Minimal instructions on data reporting were provided and linked to the lab ID, stimulation condition, mortality percentage, sample ID, investigated marker and counted spots.

#### Pilot study 2

In pilot study 2, the variation in spot interpretation and data reporting was investigated. An ELISpot plate prepared by one partner (University of Oxford) was distributed to 6 other partners for read-out within 30 days. Storage and transport of this plate were performed at room temperature. Each lab was asked to read the plate shortly after reception according to their in-house procedure, report the data according to minimal instructions provided by the organizing center, and send the plate to the next lab according to the predefined distribution schedule.

Results from both pilot studies and information provided *via* the online worksheets were collected and centrally analyzed by the organizing center (Center for Vaccinology, Ghent University, Belgium). The processed data is reported in a blinded manner in this study.

### IFN-γ ELISpot assay procedure and antigen titration

For the pilot studies, the consortium partners were asked to use their in-house procedures and preferred reagents. During assay qualification experiments performed by one FLUCOP partner, the SOP for IFN-γ ELISpot developed by FLUCOP was applied. This procedure is available as **Supplementary Material (Appendix 2)**. Briefly, the FLUCOP IFN-γ ELISpot assay was performed using the human IFN-gamma ELISpot PRO kit (3420-2APW-2, Mabtech). On the first day of the 2-day procedure, the pre-coated plates were re-hydrated by washing 4 times with PBS 1X and were blocked by adding 200 µL culture medium (RPMI supplemented with penicillin/streptomycin, L-glutamine, sodium pyruvate, MEM, ß-mercaptoethanol, FBS). Plates were stored for 2 to 4 hours at 37°C. Meanwhile, the samples were thawed in a water bath at 37°C and washed in culture medium. The cell suspension was centrifuged and cells were washed again in culture medium supplemented with benzonase. After centrifugation, cells were resuspended in culture medium. Cell concentration was measured using a Sysmex hematology analyzer (XN-L-350) and cell viability was determined with propidium iodide (PI; staining of dead cells) using a flow cytometer. After centrifugation, cells were resuspended in culture medium to obtain a concentration of 4 x 10^6^ PBMC/mL. The blocking solution was removed from the assay plates and 100 µL stimulation reagent was added to each well. PBMC were plated by adding 200 000 PBMC per well (in 50 µL). The plates were stored overnight (18-24 hours) in an incubator at 37°C and 5% CO_2_. The next day, cell suspensions were removed and plates were washed 5 times with 200 µL lab-grade water per well. Next, 100 µL of detection antibody was added and the plates were incubated for another 2 hours at room temperature. Plates were washed 3 times with 200 µL/well lab-grade water, followed by 3 more washes with 200 µL/well PBS. Substrate (50 µL) was added to each well to visualize the spots. Plates were stored at room temperature and were air-dried in the dark. Spots were enumerated within 7 days after the start of the assay using an automated spot counter (ImmunoSpot^®^ S5, Cellular Technology Limited), followed by manual verification of the identified spots (Quality Control).

The influenza-specific stimulating agent selected for the assay qualification experiments was split A/California (H1N1) virus (batch FA593899), kindly provided by Sanofi. Antigen was titrated to determine the optimal stimulation concentration (**Supplementary Figure 1**) and investigate any potential cellular toxicity. A final concentration of 1.25 µg/mL was found to be the most appropriate stimulating condition. At this concentration, no cellular cytotoxicity was observed (data not shown) and therefore, this concentration was selected for further PBMC stimulations in ELISpot assays. In some qualification experiments, tetanus toxin (T3194-25UG, Sigma-Aldrich) was used as a control antigen to stimulate the cells *in vitro* at a final concentration of 2 µg/mL. The mock or background condition consisted of PBMC cultured in medium only. Each condition was tested in triplicate and mean responses were reported.

### Data analysis

Results from the IFN-γ ELISpot assay were expressed as Spot Forming Units (SFU) per million PBMC and reported as the mean of triplicates, duplicates or as singletons, depending on the lab and/or test condition. For the evaluation of antigen-specific responses, background-subtracted results were used. Negative values were corrected to 1 SFU/10^6^ cells. Results from the pilot studies and the specificity experiment were log-transformed prior to further calculations. Individual lab results were compared to the geomean, descriptive statistics were performed and a Z-score was attributed.

The Z-score expresses the relationship of a reported value to the mean of a group of values in terms of standard deviations and shows where that result is positioned in the distribution of all reported values. The Z-score is therefore a good tool to assess the performance of an individual lab within a group. Still, it does not give information on the total imprecision of the group data. A Z-score of 0 indicates that the reported result is identical to the mean result. A Z-score of 1 indicates that the reported result differs one standard deviation from the mean. Z-scores can be positive or negative, with a positive value indicating the score is above the mean and a negative score indicating it is below the mean.


$$\text{z}=\frac{\text{x}-\text{μ}}{\text{σ}}$$

μ=mean; σ=standard deviation. As an arbitrary rule of thumb, an absolute Z-score ≤1 can be considered as excellent, a Z-score be-tween 1 and 2 as acceptable and a Z-score >2 should be considered as poor performance requiring further investigation.

## Results

### Pilot study 1

**Figure 1:**
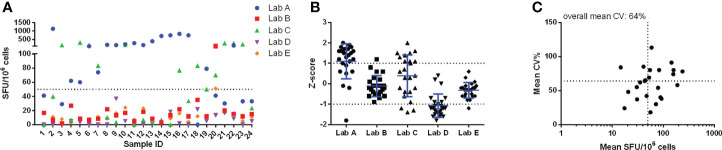
Pilot study 1 - unstimulated conditions. Results from each of the 24 samples reported by the 5 labs are given in panel **(A)** and are expressed as SFU/10^6^ cells. The performance of the 5 labs is shown in panel **(B)** and is expressed as Z-scores. Panel **(C)** shows the imprecision, expressed as CV%, for the within-sample mean values, expressed as SFU/10^6^ cells. Here the horizontal dotted line represents the overall mean CV observed (64%) and the vertical dotted line indicates the arbitrary acceptance criterion of 50 SFU/10^6^ cells. Calculations for data shown in panels **(B)** and **(C)** were performed with log-transformed data.

In pilot study 1, a large variation was already observed in the unstimulated conditions (**Figure 1**). Two out of 5 labs reported background responses exceeding 50 SFU/million PBMC in more than half of the samples (**Figure 1A**). This 50 SFU/million PBMC threshold is a commonly applied acceptance or quality criterion (11–13). This large variation is reflected in the Z-scores (**Figure 1B**) where lab A generally reported higher responses, lab D lower responses, lab C showed a dispersed profile and labs B and E reported values close to the group mean (Z-scores between -1 and 1). The overall mean coefficient of variation (CV) of the background responses was 64% (**Figure 1C**).

**Figure 2:**
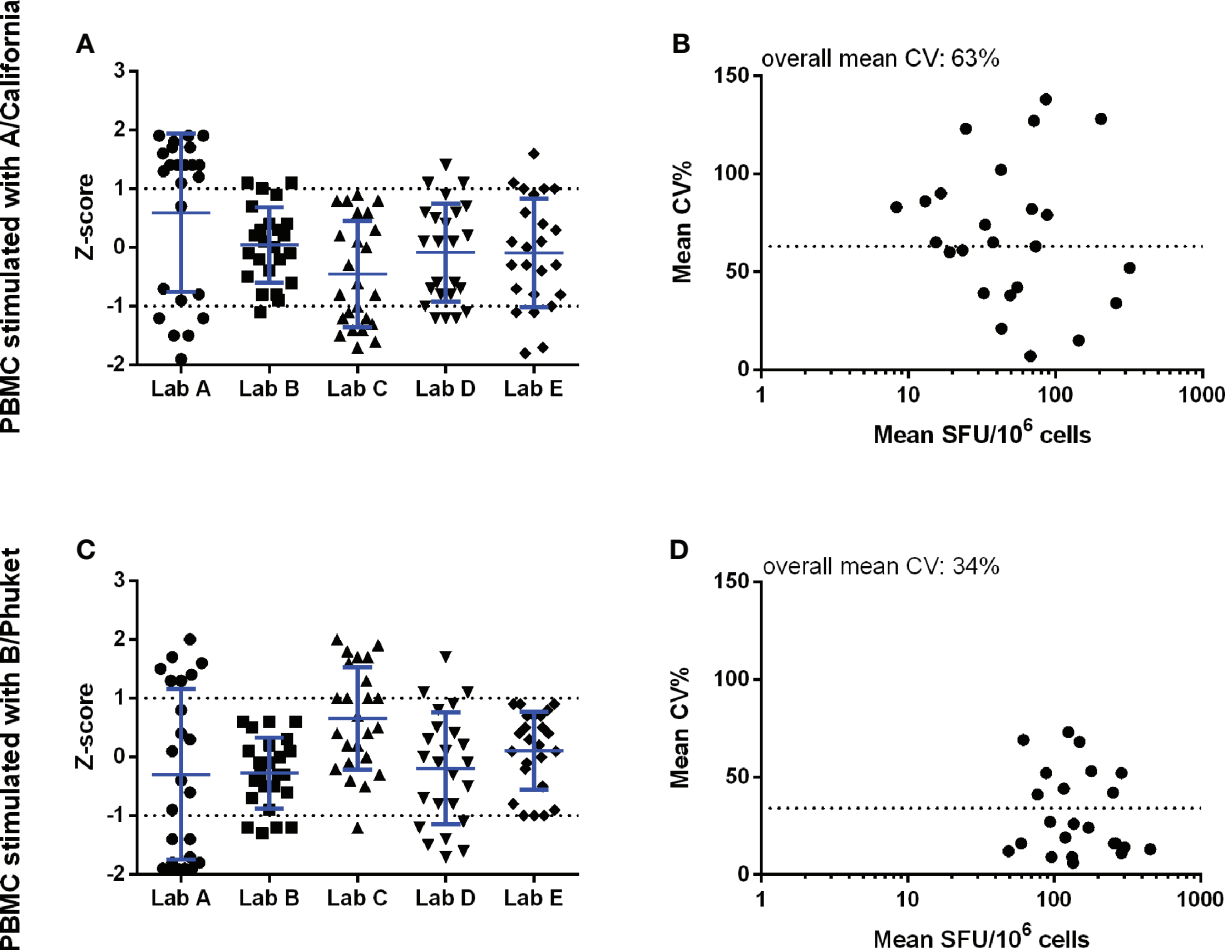
Pilot study 1 – Background-subtracted results from the cell cultures stimulated with inactivated split A/California (panels **A, B**) and B/Phuket (panels **C, D**). The performance of the 5 labs is shown in the left panels and is expressed as Z-scores. The observed imprecision, expressed as CV%, for the mean within-sample values, expressed as SFU/10^6^ cells, is shown in the right panels. Here the dotted horizontal line indicates the overall mean CV of 63 and 34% for A/California and B/Phuket, respectively. Analyses were performed with log-transformed counts.

Significant variation was also observed in the antigen-stimulated cultures. The Z-scores demonstrated that all labs reported values relatively close to the group mean, in other words, no lab generated data deviating with more than 2 standard deviations from the group mean. However, the imprecision, expressed as the mean CV, of cultures stimulated with A/California and B/Phuket demonstrated high levels of variation, with 63% and 34% CV, respectively (**Figure 2**). The lower mean CV upon stimulation with B/Phuket can be explained by the higher range of responses observed, 48.9-2069 SFU/million PBMC, whereas A/California elicited responses in the range of 8.3-319.9 SFU/million PBMC.

### Pilot study 2

**Figure 3:**
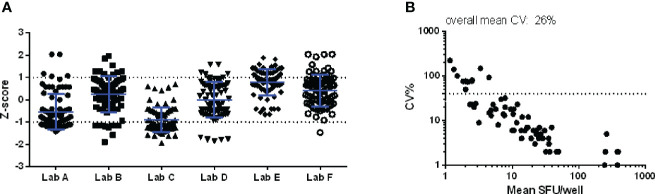
Pilot study 2. An IFN-γ ELISpot plate with 84 wells to be scored was distributed to 6 laboratories within one month after preparation. Z-scores per well per lab **(A)** and the CV per well **(B)** are shown. The dotted line in the right panel indicates the CV of 40%, commonly applied as a threshold of acceptable variation in CMI assays.

In pilot study 2, the variation that can be induced upon the enumeration of IFN-γ secreting cells by interpreting developed spots was examined. **Figure 3** shows that the Z-scores of the reported counts varied between -1 and 1 (panel A) and that the highest CV values were observed in the lower part of the analytical range (< 5 SFU/well or 25 SFU/million PBMC; panel B). Although limited variation was observed in the enumeration of the spots (mean CV of 25.8%), high diversity in reporting conditions with spots too dense to count was noticed. Some labs reported these particular conditions with a symbol (* or °), one lab with a code (“-2”), one lab with an abbreviation (TNTC, too numerous to count) and two labs with zero (“0”). This observation indicates that careful thought should be given to how such a condition should be flagged and reported. Especially the zero (“0”) or “-2” codes can lead to misinterpretation when evaluating (background-subtracted) results as this will still generate a (negative) number. It is important to clearly distinguish non-numerical values (e.g. in case of too dense spots preventing accurate counting) from numerical results (i.e. actual data). A harmonized reporting code needs to be defined.

### Establishment of the FLUCOP SOP

Based on information collected *via* the online worksheet (pilot study 1) and a thorough comparison of all applied procedures, a set of potential critical parameters was identified. These were: the cell count technique, application and duration of a cell resting phase, the use of self- versus pre-coated plates, the concentration of cells in the wells, the time of incubation, the medium used during stimulation, the wash step, the ELISpot reader and the FBS validation status (**Table 1**).

**Table 1:** Overview of the parameters that were identified as potentially critical for the outcome of the IFN-ɣ ELISpot assay.

Lab ID	Cell count technique	Cell resting	Duration of cell resting (hours)	Plates	Concentration of cells in wells	Incubation time (hours)	Culture medium used in cell resting and during stimulation	Wash step	ELISpot reader	FBS validation
Lab 01	Automated	Yes	4	Self-coated	200.000	18	cRPMI	Automated	AID-ISpotSpectrum	Validated
Lab 02	Automated	No	No	Self-coated	500.000	22	cRPMI	Manually	ImmunoSpot S6, CTL	Validated
Lab 03	Automated	Yes	19	Pre-coated	300.000	24	cRPMI	Manually	ImmunoScan, CTL	No info
Lab 04	Manual	Yes	3	Self-coated	250.000	19	cRPMI	Manually	ELISpot 7.0 - iSpot	Validated
Lab 05	Automated	Yes	1,5	Pre-coated	200.000	19.5	cRPMI	Manually	ImmunoSpot S6, CTL	No
**Consensus** **protocol**	**Validated method**	**Yes**	**3**	**Free of choice**	**200.000**	**Overnight (18-24 h)**	**Complete RPMI (cRPMI)**	**Your validated method**	**Your validated equipment**	**Validated FBS**
Fixed or as recommended	Recommended	Fixed	Fixed	/	Fixed	Fixed	Recommended	Recommended	Recommended	Fixed

Within the consortium, a consensus was reached to define a set of specific parameters that had to be strictly applied in the standardized operating procedure for IFN-γ ELISpot testing, whereas for other parameters only a recommendation was proposed (**Appendix 2**).

### IFN-γ ELISpot assay qualification

#### Linearity

Linearity of an assay is its ability to provide test results that are directly proportional to the concentration of the measurand (quantity to be measured) in a test sample. A standard approach to assess the linearity of a laboratory method consists of diluting a test sample in a negative sample matrix to demonstrate then the assay’s ability to reproduce the initial result after recalculation of the result obtained after dilution. In complex cell-based assays, where no reference materials are available, this approach requires the availability of non-responding cells to dilute the cell(s) of interest. In the IFN-γ ELISpot assay, where *in vitro* stimulation occurs immediately prior to the read-out and in the same final reaction vessel, this would require that the diluting cells do not induce any allogeneic or bystander effect on the “tested cells”. As this cannot be achieved, an adapted method was designed.

The linearity of the IFN-γ ELISpot was assessed using 4 PBMC samples that were independently fractionally diluted in culture medium without (background condition) or with split A/California virus as stimulating antigen. This was done in 3 replicates and repeated 3 (sample 4) or 4 (samples 1-3) times. Fractional dilution of the samples was done by plating 40 000 to 400 000 cells per well. The microculture with 200 000 cells per well was selected as the reference condition for calculating the recovered response.

At this condition of 200 000 cells per well, 127.8 – 15.3 – 15.5 and 13.6 SFU/well were counted in samples 1, 2, 3 and 4, respectively (**Table 2**). Expressed as SFU per million PBMC, this translated into 639.2 – 76.7 – 77.5 and 67.9 SFU for samples 1, 2, 3 and 4, respectively.

**Table 2:** Observed SFU per well at each dilution and recovery.

	Sample 1		Sample 2		Sample 3		Sample 4	
Number of cells plated	Observed mean SFU/well	Recovery (%)	Observed mean SFU/well	Recovery (%)	Observed mean SFU/well	Recovery (%)	Observedmean SFU/well	Recovery (%)
40 000	7.6	28.1	1.0	35.7	0.4	3.2	1.5	19.2
80 000	26.3	51.2	3.7	53.6	3.2	45.5	3.5	76.9
120 000	58.3	75.5	5.0	51.6	5.1	55.2	6.9	100.4
160 000	89.2	87.4	10.0	69.9	9.0	73.9	8.4	88.1
**200 000**	**127.8**	**100.0**	**15.3**	**100.0**	**15.5**	**100.0**	**13.6**	**100.0**
240 000	155.9	101.3	17.3	98.2	18.3	99.0	17.0	109.0
280 000	191.4	107.0	21.7	71.4	22.7	106.7	22.4	122.7
320 000	221.8	107.7	27.7	72.9	26.7	112.4	25.0	118.6
360 000	270.7	117.5	30.7	84.7	36.2	143.2	31.3	148.1
400 000	331.3	130.6	38.3	107.1	41.6	148.4	34.9	151.3

Recovery is calculated as the percentage of mean SFU/106 cells observed in the reference condition of 200 000 cells plated per well expressed as SFU/106 cells (indicated in bold).

**Figure 4:**
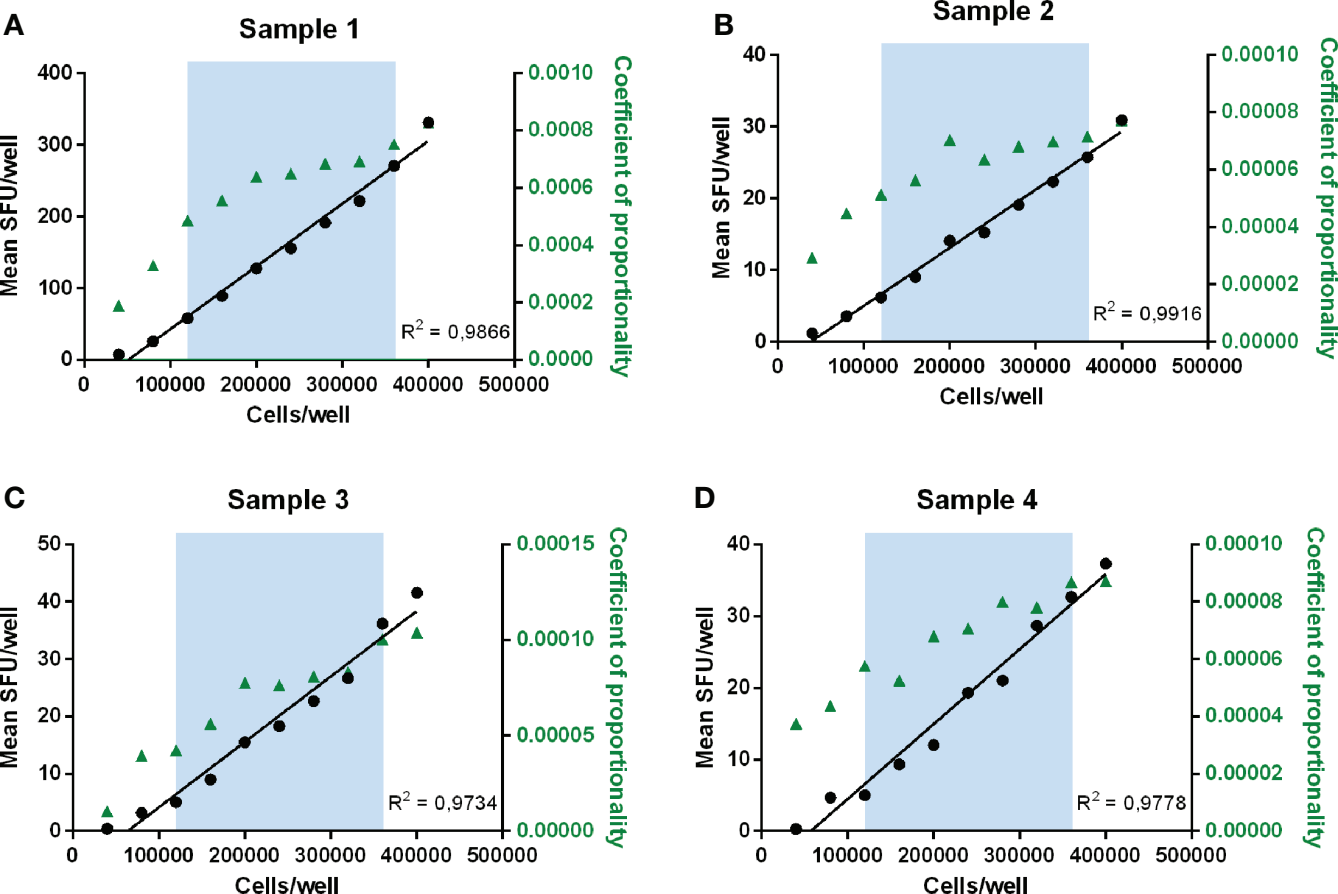
Determination of linearity and proportionality. Mean SFU/well is plotted against the number of cells plated per well for each sample **(A–D)**. The blue zone indicates the range between 120 000 and 360 000 cells/well where recovery values ranged between 50 and 150% and therefore linearity was demonstrated. Correlation curves of the number of plated cells per well and the mean responses (SFU/well) are shown in black with the concerned R2 values. The coefficients of proportionality, calculated as the ratios of the observed number of spots per well versus the number of plated cells, are indicated in green triangles and represented on the right y-axis.

As a rule of thumb, a recovery value between 50 and 150% of the observed reference value, or in other words the ability to detect a decrease or increase of the signal with 50%, is regarded as “linear”. **Table 2** clearly shows that the responses were linear within the assay conditions ranging between 120 000 cells and 360 000 cells per well. Within this technical assay range, the lowest background-subtracted response observed was 3.5 SFU/80.000 cells. The proportionality of the method was further assessed by calculating the ratios of the number of plated cells versus the observed number of spots per well. Two variables are considered proportional if their corresponding elements have a constant ratio, which is called the coefficient of proportionality. The correlation curves demonstrated high correlations in all 4 measured samples with R^2^ values ranging from 0.9734 to 0.9916 (**Figure 4**).

Within the linear range of 120 000 to 360 000 cells per well and taking into consideration the acceptable recovery range of 50-150%, the lowest and highest acceptable number of spots per well are 3.5 and 331.3 SFU. If this is extrapolated to the standard condition of 200 000 cells per well, this can be further defined as the Lower Limit of Linearity (LLOL) and Upper Limit of Linearity (ULOL) of the IFN-γ ELISpot assay executed according to the FLUCOP SOP and can be set at 25.4 and 1353.3 SFU per million PBMC, respectively.

#### Intermediate precision and repeatability

**Figure 5:**
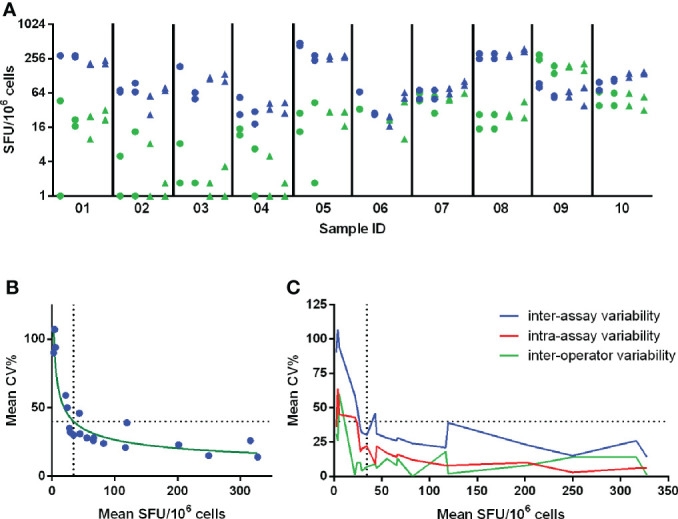
Determination of Lower Limit of Intermediate Precision (LLOIP). **(A)** Distribution of background-subtracted responses after *in vitro* stimulation of cells with A/California and tetanus toxin (TT). Each symbol represents a measurement of a certain sample, indicated on the x-axis. A set of 10 samples was tested each in duplicate by 2 operators performing each 2 runs on different days, resulting in 8 measurements per sample. Measurements from operator 1 are indicated with circles and from operator 2 with triangles. Responses obtained after stimulation with A/California are indicated in blue, with TT in green. **(B)** LLOIP was determined by plotting the mean background-subtracted responses to A/California and TT against the mean inter-assay CV% per sample. The LLOIP was defined as the lowest response with a CV of 40% and was determined at 34.4 SFU/10^6^ cells. **(C)** The inter-assay, intra-assay and inter-operator variability were assessed by the mean CV% observed for the mean responses of each sample. Each horizontal dotted line represents the cut-off of 40% CV and each vertical dotted line represents the LLOIP determined at 34.4 SFU/10^6^ cells.

The IFN-γ spot forming responses elicited by A/California and TT antigens were measured in 10 PBMC samples. Each sample was tested in duplicate by 2 operators performing each 2 runs on different days, resulting in 8 measurements per sample. The mean responses ranged from 34.4 to 327.5 SFU/10^6^ cells and from 2.5 to 201.5 SFU/10^6^ cells after stimulation with A/California and TT, respectively (**Figure 5A**). The variability was assessed by calculating the CV.

Inter-assay or intermediate precision was assessed as the average CV% of all individual measurements per sample after stimulation with TT and A/California and ranged from 14 to 107%, with a mean of 41%. The intra-assay precision or repeatability was assessed and determined across all mean responses obtained per run and ranged from 0 to 36%, with a mean of 21%. The inter-operator precision was also assessed and determined across all mean responses collected per operator for a particular test condition and ranged from 0 to 60%, with a mean of 12%. The level of variation increased substantially in the lower part of the analytical range of this assay, as shown in **Figure 5C**.

The Lower Limit of Intermediate Precision (LLOIP) was determined by plotting the mean background-subtracted responses to TT and A/California against the mean inter-assay CV% per sample (**Figure 5B**). The LLOIP was defined as the lowest value with a CV of 40%. A log-log line was considered as the best-fitted curve with an R^2^ = 0.8729 and y=10^((-0,3820*log(x))+2,189), where *y* is the mean CV% and *x* the mean SFU/10^6^ cells per sample. The lowest background-subtracted response with a CV of 40% was determined at 34.4 SFU/10^6^ cells.

The Lower Limit of Quantification (LLOQ) was defined as the highest value of the LLOL and LLOP and therefore set at 34.4 SFU per million cells. In the white paper published by Corsaro et al. (14), the acceptance criterion for the intermediate precision is defined as ≤40% for ≥80% of the samples having mean spot forming units/million cells exceeding the LLOQ. In this study, 96 out of 160 obtained values exceeded the LLOQ determined at 34.4 SFU/million cells, resulting in an intermediate precision of 20% (ranging from 4 to 39%).

#### Specificity

**Figure 6:**
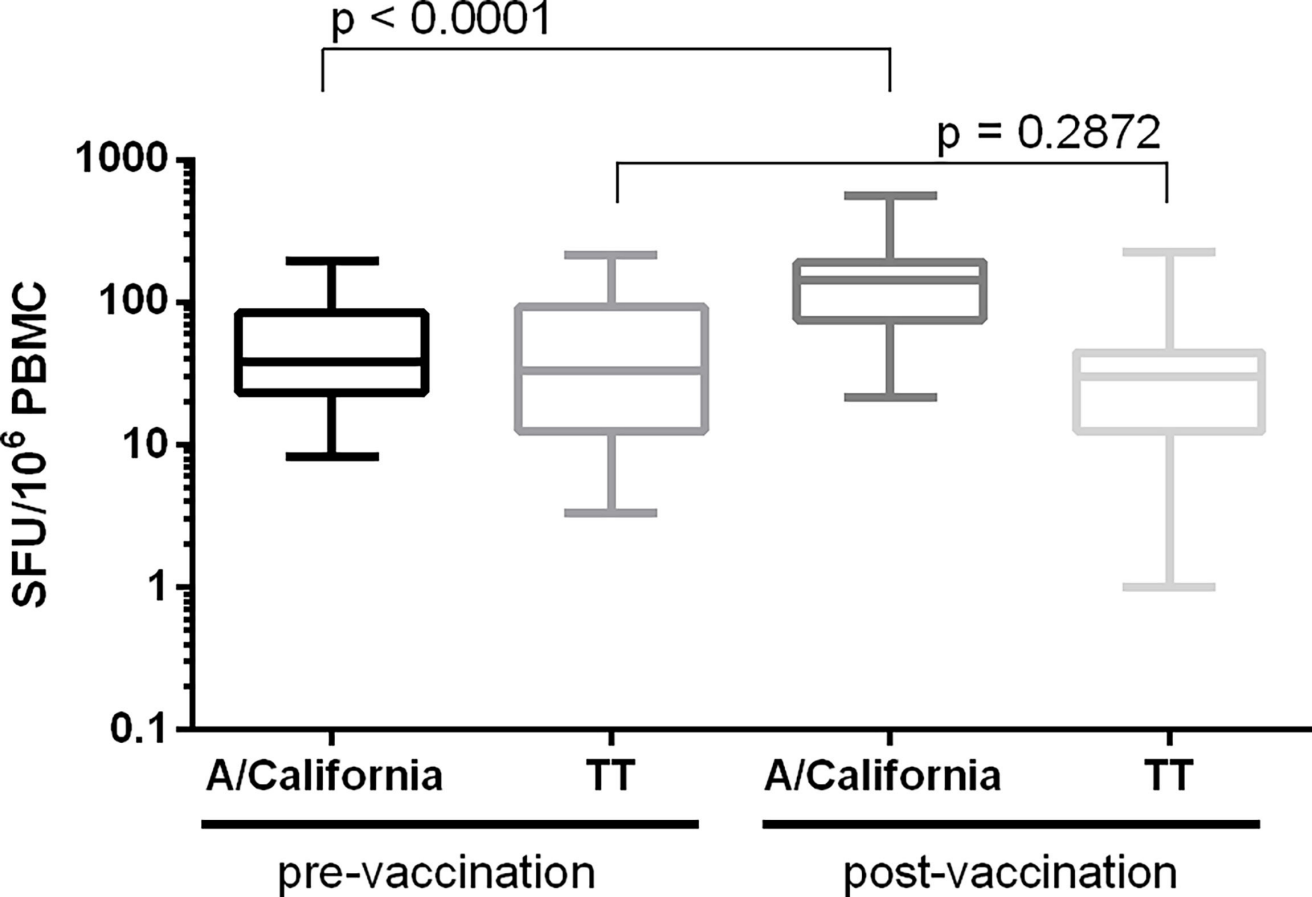
Determination of assay specificity. PBMC from 25 paired samples collected before and 7 days after the administration of a seasonal influenza vaccine were stimulated with influenza (split A/California) and control (tetanus toxin; TT) antigens. Background-subtracted values are shown as box plots. Differences in IFN-γ spot forming responses between the pre- and post-vaccination samples following *in vitro* stimulation with either influenza or TT antigen were examined. Wilcoxon matched-pairs signed-rank test was applied and p < 0.05 was considered statistically significant.

Assay specificity was determined by stimulating PBMC from 25 paired samples collected before and 7 days after the administration of a seasonal influenza vaccine. PBMC were stimulated with split A/California virus and tetanus toxin, a control antigen unrelated to the vaccine. Specificity was demonstrated if a significant increase in signal was observed between the pre- and post-vaccination samples upon stimulation with the selected influenza-specific antigen (p< 0.05) and if no significant increase was observed after stimulation with the control antigen (p ≥ 0.05). **Figure 6** shows a significant increase of the influenza-specific response (p < 0.001; Wilcoxon signed-ranked test) and no difference in the TT-specific response (p = 0.2872, Wilcoxon signed-rank test).

#### ULOQ

**Figure 7:**
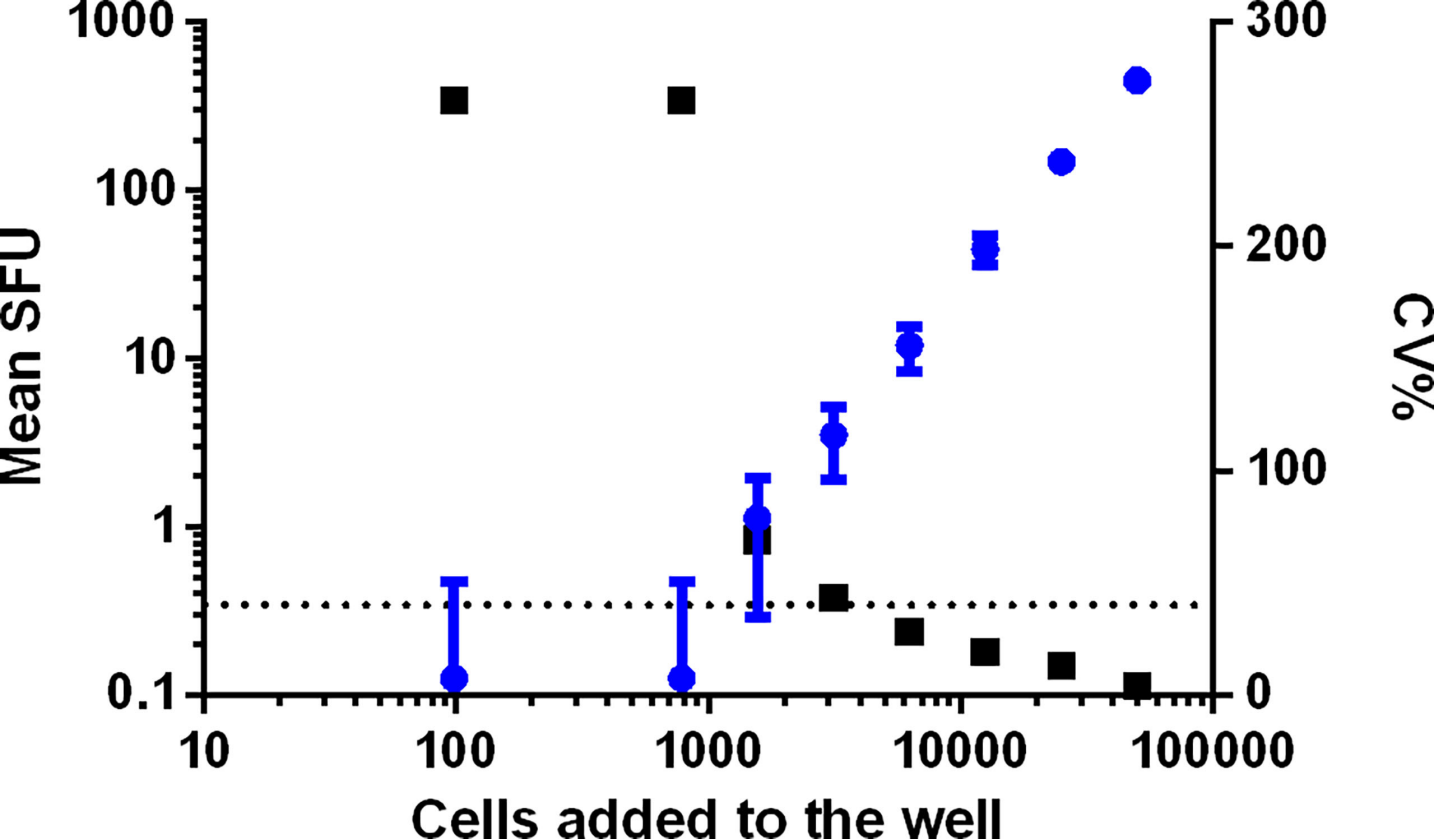
Determination of the ULOQ. PBMC from two samples were plated each on one plate in a serial dilution, ranging from 100 000 to 48 PBMC per well. Data from sample 1 is shown. Each condition was repeated 8 times and all cells were stimulated with SEB. Mean SFU with SD bars is indicated in blue and represented on the left y-axis. The related CV% are shown in black and represented on the right x-axis. The dotted line indicates the acceptance criterion of 40% CV.

PBMC from two samples were plated each on one plate. The cells were added to the plates in a serial dilution ranging from 100 000 to 48 cells, with 8 repeats per dilution. All wells were stimulated with the superantigen SEB (staphylococcal enterotoxin B) at a final concentration of 0.25 µg/mL. The Upper Limit of Quantification (ULOQ) is the highest number of counts per well that can be detected and reliably quantified, i.e., a CV% ≤ 40. For both samples, reliable counts were reported up to and including the test condition where 50 000 PBMC were stimulated with SEB with CVs of 4 and 6% **Figure 7** (sample 1) and **Supplementary Figure 2** (sample 2). The wells seeded with 100 000 PBMC generated too many spots preventing an accurate spot count. The determination of the ULOQ is primarily defined by the instrument and the operator(s) and depends on the availability of strong positive samples. The highest responses that could be enumerated accurately in this analyzing lab were 452.25 and 458.75 SFU/50 000 PBMC or 9045 and 9175 SFU/10^6^ PBMC. Based on this information, it can be proposed that samples or conditions resulting in too many spots are reported as ‘> 10 000 SFU/10^6^ PBMC’.

#### Limit of detection (LOD)

The Limit of Detection (LOD) is the lowest detectable analyte concentration that can be reliably distinguished from analytical noise (15). The technical LOD is calculated as the 95^th^ percentile of the non-specific responses after mock stimulation (medium only). Based on 402 values from the linearity, intermediate precision, repeatability and specificity experiments, the LOD was determined at 12.70 SFU/well or 63.50 SFU/million PBMC. This cut-off could be applied as a criterion of acceptance for background responses instead of the commonly used arbitrary albeit more stringent cut-off of 50 SFU/million PBMC.

#### Validity of signal stability over time

**Figure 8:**
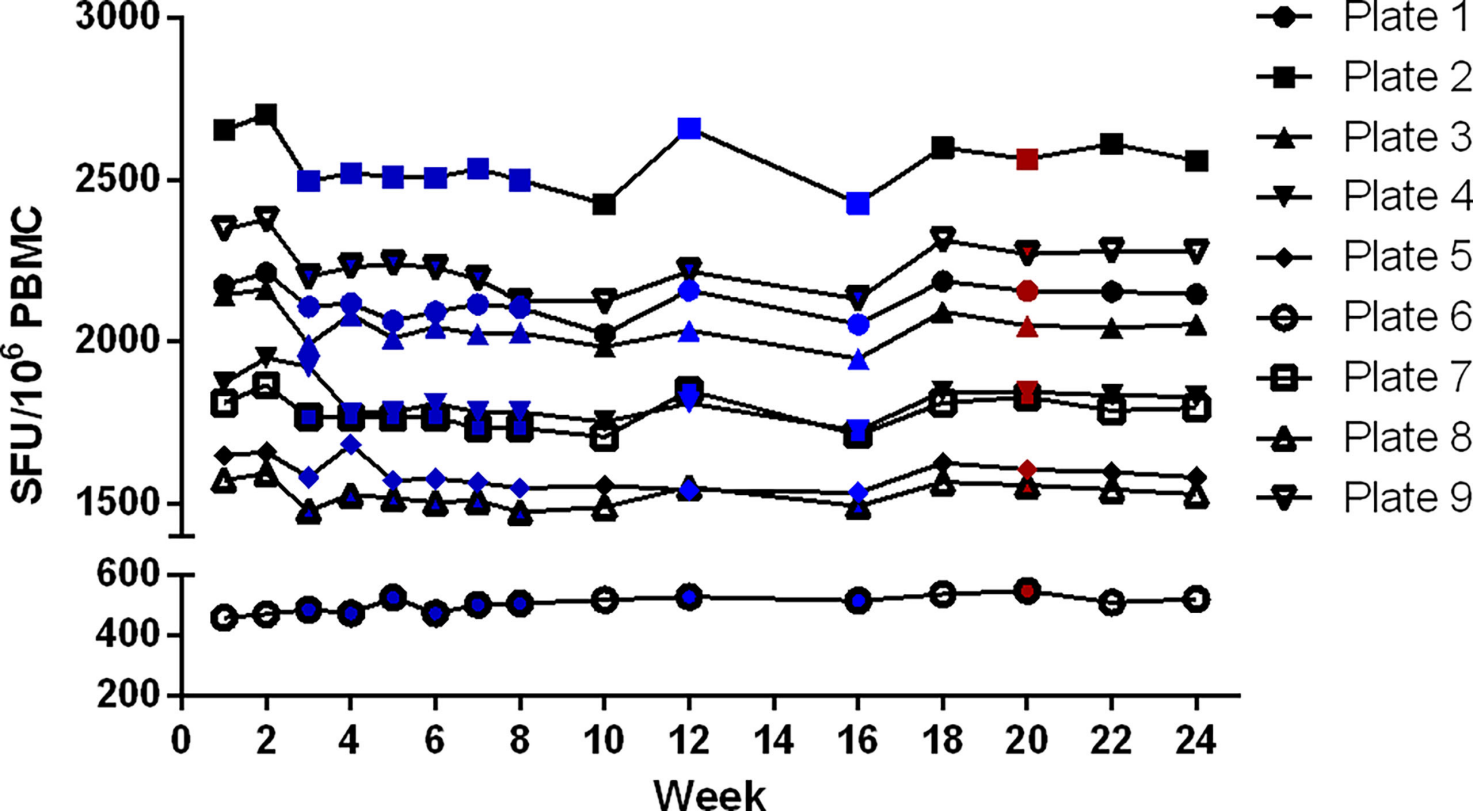
Determination of validity of signal stability over time. A set of 9 plates was re-read every other week for 24 weeks. The sum of all antigen-specific IFN-γ ELISpot responses of each plate was evaluated over time. Reading of the plates was performed by 3 different operators, indicated in black (n = 6), blue (n = 8) and red (n = 1).

A set of 9 developed plates was re-read every other week for 24 weeks. The stability of the signal was demonstrated as the counts did not change significantly during this period (**Figure 8**). The total counts of 96 wells per plate varied with CVs between 2 to 5%, with a decrease of the total sum of counts in 8 out of 9 plates compared to the first read (-1 to -4%) and one plate with an increase of 14%. The absence of striking differences in the reported values for these 3 different operators appears to be an indication of a low inter-operator variability. It is assumed that all involved operators were equally qualified in spot enumeration and because of the limited number of reads per operator this was not further examined. The effect of time was statistically analyzed by performing one-way ANOVA tests. The tests demonstrated a statistically significant effect of time as of week 7 (p < 0.0001), meaning that the plates remain stable and can be stored up to and including 6 weeks after development.

A summary of all qualification results of the IFN-γ ELISpot assay is shown in **Table 3**.

**Table 3:** Assay qualification summary.

Assay parameter	Acceptance criteria	Qualification outcome
Intermediate precision and repeatability	The CV for reproducibility should be ≤ 40.% (*The CV for reproducibility should be ≤ 40% for ≥80% of the samples having SFU/10^6^ PBMC > LLOQ.) The Lower Limit of Intermediate Precision is defined by the lowest measurement with a CV of 40%.	Stimulation with A/California: Inter-assay CV is 27% (*20%) Intra-assay CV is 10% (*8%) Inter-operator CV is 8% (*8%) Stimulation with TT: Inter-assay CV is 55% (*19%) Intra-assay CV is 31% (*14%) Inter-operator CV is 17% (*6%) Pooled data: Inter-assay CV is 41% (*20%) Intra-assay CV is 21% (*9%) Inter-operator CV is 12% (*7%) LLOIP = 34.4 SFU/10^6^ PBMC
Linearity	The range of cell densities (plated cells) through which the recovery was between 50 and 150%.	Linear range: from 120 000 to 360 000 cells/well
LLOQ	The highest value observed of the Lower Limit of Precision and Lower Limit of Linearity.	LLOQ = 34.4 SFU/10^6^ PBMC
Specificity	Comparison of pre- and post-vaccination samples: Significant increase after influenza-specific stimulation of the cells (p < 0.05) AND Non-significant increase after stimulating the cells with control (non-vaccine) antigen (p ≥ 0.05)	Stimulation with A/California: p < 0.0001 Stimulation with TT: p = 0.7260
ULOQ	The highest number of counts per well that can be detected and reliably quantified, i.e. with a CV% ≤ 40.	ULOQ = 458.75 SFU/50 000 PBMC
LOD	The 95^th^ percentile of the responses after mock stimulation.	LOD = 12.70 SFU/well or 63.50 SFU/million PBMC
Signal stability over time	No significant waning of counts over time.	No significant effect of time up to and including 6 weeks after development (p ≥ 0.0001).

## Discussion and conclusion

The IFN-γ ELISpot and intracellular cytokine staining (ICS) assays are frequently used to examine cellular immune responses elicited by influenza infection or vaccination (16–21). A better insight into the magnitude, quality and durability of cell-mediated immune responses can improve our understanding of the immunological mechanisms underlying viral clearance or vaccine effectiveness and may contribute to the identification of new correlates of protection. The first prerequisite to generate reliable data is the quality of the samples to be studied. To produce high-quality PBMC, it is desirable to apply a standardized procedure for the isolation and cryopreservation of PBMC that has been proven to be compatible with the envisaged downstream analyses (22–25). Secondly, laboratories that monitor cellular immune responses with the IFN-γ ELISpot assay apply a variety of in-house procedures. This generates diverging results, complicates interlaboratory comparisons, and hampers a reliable evaluation of the immunogenicity of influenza vaccines (4–6). For these reasons, there is still a great need to harmonize the procedures and provide guidance on how to report the assay results in a standardized manner. Within the European FLUCOP consortium, we first developed SOPs for influenza-specific IFN-γ ELISpot (described in this paper) and ICS assays (published in *Begue et al. 2022. Harmonization and Qualification of Intracellular Cytokine Staining to Measure Influenza-Specific CD4^+^ T Cell Immunity Within the FLUCOP consortium (submitted))* and subsequently performed qualifications of the assays.

Two pilot studies performed in 5 to 6 labs allowed us to identify critical and less critical parameters that can induce assay variation. When every lab applied its in-house procedure, the observed variation, expressed as the coefficient of variation, was 64% in the unstimulated conditions and 63 and 34% in the cell cultures stimulated with split A/California and B/Phuket virus, respectively. Lower variation (mean CV of 25.8%) was observed when only the spot enumeration was assessed. However, high diversity was noticed in conditions where the spots were too dense to count. Based on the reported data and observed ULOQ, ‘> 10 000 SFU/10^6^ PBMC’ is proposed as a harmonized reporting code. The overall heterogeneity observed in both pilot studies was considered modest. This may be due to the use of commercial IFN-γ ELISpot kits by several laboratories, a very advantageous possibility not available for all immunoassays. Several assay variables were identified as potential critical parameters: the cell counting technique, the use and duration of a cell resting phase, the use of self- versus pre-coated plates, the cell number per well, the incubation time, the culture medium during stimulation, the automated or manual execution of wash steps, the ELISpot reader and the validation status of the FBS. Based on the information collected during the pilot studies, a consensus was reached and a standardized operating procedure for IFN-γ ELISpot testing protocol was developed that consisted of a set of specific parameters that had to be strictly applied, whereas for some additional conditions only a recommendation was proposed. Strict application was required for a cell resting phase (3 hours), seeding density of 200 000 cells per well, an overnight incubation period defined as from 18 to 24 hours and the use of validated FBS. Having a resting phase of cells prior to addition to the ELISpot plate is considered advantageous (26). Cells in an apoptotic state upon thawing will die during the resting phase, and therefore, the proportion of living and good-quality cells will increase leading to a more correct number of plated cells. The number of cells added to a well was also considered a crucial parameter. A single layer of cells is typically achieved by adding 100 000 to 150 000 cells per well. A higher concentration of cells/well can be beneficial for a more effective antigen presentation and co-stimulation. However, an excessively high concentration can lead to spot crowdedness and elevated background spots, which negatively impact the assay sensitivity (27). The latter was observed in pilot study 1 when 500 000 cells per well were seeded by lab A. The consensus was reached to add 200 000 cells/well in the FLUCOP SOP, which is in line with several other publications (28–30). The overnight incubation step was further defined as a period of 18 to 24 hours in line with what the FLUCOP partners were performing. Finally, the use of pre-screened and validated FBS was considered critical because this assay component may cause spontaneous, non-specific cytokine secretion that may have a significant impact on assay sensitivity or may contain toxic factors that can dampen antigen-specific responses.

This harmonized SOP for IFN-γ ELISpot testing was then subjected to a qualification process performed by one FLUCOP partner (CEVAC, Ghent University and University Hospital, Belgium). Unlike for immunoassays such as ELISA, no universally accepted procedures are available to qualify or validate ELISpot assays for regulated use. General criteria that can be applied to define the linearity, intermediate precision, repeatability, LLOQ, ULOQ, LOD and signal stability over time are also lacking. Here we describe the qualification of an IFN-γ ELISpot assay in which human PBMC were stimulated with influenza and tetanus toxin antigens. The acceptance criteria applied to the assay characteristics mentioned above were based on what was available in literature and white papers (14, 15, 28, 29, 31, 32). A summary of all qualification criteria and results is shown in **Table 3**. The acceptance criterion for reproducibility was set at a CV of ≤ 40%, a cut-off commonly applied in cell-based assays (14, 28). In a first analysis of the data, all values were taken into account to determine the inter-assay variability or intermediate precision. However, a recently published white paper recommended to exclude values below the LLOQ for this calculation (14). These lower counts greatly impact the level of CV because of the higher imprecision in that part of the analytical range of the assay. The observed intermediate precision as of the LLOQ was estimated at 20% and demonstrated the robustness of this assay. The remaining variables responsible for divergent assay results, but more difficult to harmonize, are for example the cell counting techniques and viability measurements, various reagents other than FBS, the ELISpot reader, the spot identification settings that are applied, and the level of experience of the operators. The lack of appropriate reference standards and positive control samples, especially those that mimic test samples, represent additional challenges. Finally, a harmonized protocol itself does not guarantee good performance. Proper execution of a protocol requires skills as well as appropriate training and needs regular quality assessment, not only within a lab but also between labs by conducting interlaboratory or proficiency tests.

Assay qualification is a first step towards assay validation and provides already a means to ensure that the generated data are credible and reproducible. Even in research settings and non-regulated laboratories, this can provide valuable information on the fitness of the assay for the intended use and how to interpret the data (signal versus noise). It is recommended to test a variety of antigens before assay qualification is initiated to define the most suitable stimulating agent compatible with the assay and to answer the appropriate research questions. Furthermore, it is essential that the assay is qualified with the antigen that will be used in the final analysis. If there is a need to change the type of antigen, then a requalification of the assay could be required. However, a change of antigen lot can be supported by performing a bridging experiment without repeating any or all of the qualification experiments, but this should have been addressed by a robustness experiment during assay qualification.

In conclusion, the FLUCOP harmonized IFN-γ ELISpot assay procedure can accurately enumerate IFN-γ secreting cells in the analytical range of 34.4 SFU/million cells up to the technical limit of the used reader and in the linear range from 120 000 to 360 000 cells per well, in plates stored up to 24 weeks after development. We hope this harmonized IFN-γ ELISpot procedure will become a useful and reliable tool to investigate influenza-specific cellular immune responses induced by natural infection or vaccination and will be an aid in the search for novel correlates of protection. We estimate that this harmonized assay may also be applied to cellular immune responses against other (respiratory and non-respiratory) infectious pathogens.

## Data availability statement

The raw data supporting the conclusions of this article will be made available by the authors, without undue reservation.

## Ethics statement

The studies involving human participants were reviewed and approved by the Ethical Committee of the Ghent University Hospital. The patients/participants provided their written informed consent to participate in this study.

## Author contributions

FC, GL-R, and GW contributed to the conception and design of the study, data analysis and interpretation, and wrote the first draft of the manuscript. TL, DB, FC, GL-R, and GW contributed to the data acquisition. RC contributed to the critical revision of the manuscript. All authors have read and approved the final version for submission.

## Funding

The FLUCOP project is supported by the Innovative Medicines Initiative-Joint Undertaking under grant agreement 115672, resources of which are composed of financial contribution from the European Union’s Seventh Framework Programme (FP/2007-2013) and EFPIA companies’ in kind contribution.

## Acknowledgments

The authors wish to thank Annelies Goussaert, Peter Vander Linden, Sanne Foubert and Sibyl Couvent for the data acquisition and Sara Tete for the critical review of the manuscript. The authors would like to acknowledge their FLUCOP consortium collaborators for their assistance: Catherine Caillet, Barbara Camilloni, Maria Rita Castrucci, Marco Cavaleri, Annalisa Ciabattini, Simon De Lusignan, Oliver Dibben, Othmar Engelhardt, Susanna Maria Roberta Esposito, Marzia Facchini, Felipa Ferreira, Sophie Germain, Sarah Gilbert, Sammy Ho, Katja Hoschler, Sarah L Jalloh, Stefan Jungbluth, Marion Koopmans, Manuela Mura, Nedzad Music, Martina Ochs, Thierry Ollinger, Albert Osterhaus, Anke Pagnon, Giuseppe Palladino, Elena Pettini, Ed Remarque, Leslie Reperant, Hanna Sediri Schön, Sarah Tete, Alexandre Templier, Claudia Trombetta, Serge van de Witte, Ralf Wagner, Joanna Waldock, Brenda Westerhuis, Fan Zhou.

## Conflict of interest

Authors EM and EG are employed by VisMederi srl.

The remaining authors declare that the research was conducted in the absence of any commercial or financial relationships that could be construed as a potential conflict of interest.

## Publisher’s note

All claims expressed in this article are solely those of the authors and do not necessarily represent those of their affiliated organizations, or those of the publisher, the editors and the reviewers. Any product that may be evaluated in this article, or claim that may be made by its manufacturer, is not guaranteed or endorsed by the publisher.
