# Supplementary material for: Harmonization and qualification of an IFN-γ Enzyme-Linked ImmunoSpot assay (ELISPOT) to measure influenza-specific cell-mediated immunity within the FLUCOP consortium
Source: Front Immunol. 2022 Sep 8;13:984642. doi: 10.3389/fimmu.2022.984642 (PMC9493492; doi:10.3389/fimmu.2022.984642)
Supplement: Supplementary file 1 [file DataSheet_1.docx]

#
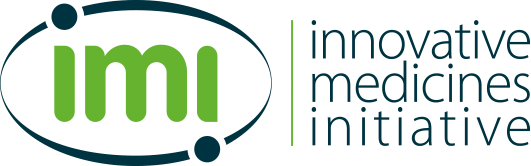


**
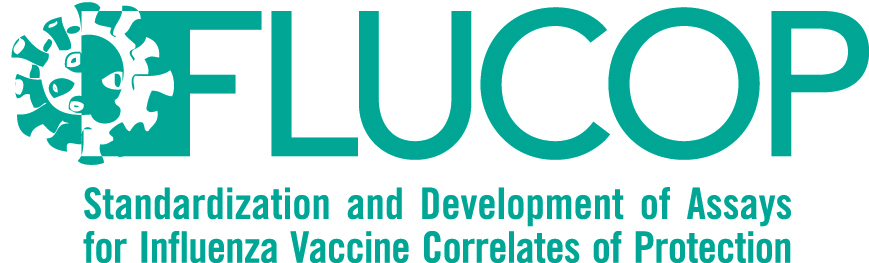
**

**Standard Operating Procedure for interferon-gamma ELISpot**

Table of Contents

[1](#_Toc106960408)

[INTRODUCTION 3](#_Toc106960409)

[REAGENTS 4](#_Toc106960410)

[MATERIALS and EQUIPMENT 4](#_Toc106960411)

[TRAINING AND PROFICIENCY 4](#_Toc106960412)

[PROCEDURE 5](#_Toc106960413)

[ELISpot procedure Day 1 - Thawing of PBMCs 5](#_Toc106960414)

[ELISpot procedure Day 1 - Preparation of stimuli 6](#_Toc106960415)

[ELISpot procedure Day 1 - Cell stimulation 6](#_Toc106960416)

[ELISpot procedure Day 2 7](#_Toc106960417)

# INTRODUCTION

The overall objective of Work Package 2 (WP2) within the FLUCOP project was to advance the understanding and application of cell-mediated immunity assays as tools for evaluating the immunogenicity of influenza vaccines. One of the tasks was to create a standardized protocol for executing an interferon-gamma (IFN-γ) Enzyme-Linked ImmunoSpot (ELISpot) assay.

The ELISpot assay allows the detection and enumeration of IFN-γ-secreting cells at the single-cell level. Peripheral blood mononuclear cells (PBMC) are stimulated with influenza antigens and are transferred to a microplate precoated with IFN-γ-specific antibodies. During an incubation period, secreted IFN-γ will be captured by the IFN-γ-specific antibodies coated on the surface. After washing away cells and unbound substances, a biotinylated antibody specific for IFN-γ is added to the wells. Following a wash to remove any unbound biotinylated antibody, alkaline-phosphatase conjugated to streptavidin is added. Alternatively, detection antibodies directly conjugated to an enzyme are added. Unbound enzyme is subsequently removed by washing and a substrate solution is added. Dark-colored precipitates will be formed and appear as spots at the sites of cytokine localization. Each individual spot represents an individual IFN-γ-secreting cell. The spots can be counted visually or with an ELISpot reader system.


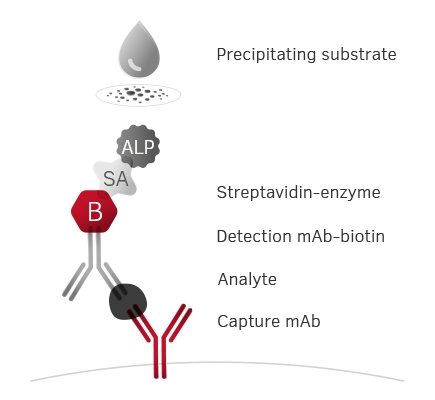


Afbeelding 1. Schematic illustration of the principle of the ELISpot assay.
(source: <https://www.mabtech.com/sites/default/files/elispot-assay-principle_0.jpg>

Although most laboratories perform the ELISpot assay using a commercially available kit, several variables can have an impact on the outcome of this assay. To examine which parameters could influence the results, an interlaboratory comparison was conducted in the context of the FLUCOP project. In total five laboratories participated and performed the assay according to their in-house protocol with samples and stimulating agents provided by the project coordinator. The main conclusions of this experiment were that although most of the labs produced comparable ELISPOT results, further harmonisation and validation would increase interlaboratory precision and improve its performance in interlaboratory studies. This protocol provides guidelines and recommendations to assist laboratories to generate accurate and reproducible ELISpot results.

#

# REAGENTS

Reagents are critical in the ELISpot assay. It is important to work with high-quality PBMC samples (high viability). The tissue culture media (complete RPMI or cRPMI) need to contain sufficient nutrients to safeguard the structural and functional integrity of the cells and allow them to respond adequately to activation signals. Foetal bovine serum should be validated before use to check whether it provides sufficient support without generating undesirable background noise. Buffers should be prepared using lab-grade water only.

It is good practice to trace the lot numbers of the used reagents, their expiration dates and certificates of analysis.

# MATERIALS AND EQUIPMENT

Using good quality lab materials will ensure a good quality of the processed sample. Plastic disposables need to be of lab-grade quality and sterile (packaged individually). Changing suppliers or product brands should trigger a “change control process” to avoid accidental misfortune and subsequent sample loss. Equipment qualification is crucial for the success of the process. All instruments should be well designed and fully qualified (based on the method’s suitability and established standards) before taking into production. Proper control, maintenance and requalification after technical intervention should assure good performance throughout their lifecycle.

# TRAINING AND PROFICIENCY

It is good practice first to train the technician and obtain proof of their proficiency before engaging the technician in conducting ELISpot assays for clinical trials purposes. Especially counting the spots requires skilled and experienced personnel.

# PROCEDURE

| General recommendations:  FLUCOP recommends executing the IFN-γ ELISpot for monitoring of cellular immune responses in the context influenza vaccine trials under the control of a proper Quality Assurance program.  Available guidelines are:   - ICH E6 R2 Guidance for Industry, Good Clinical Practice - Good Clinical Laboratory Practice (GCLP) Version 2, BARQA, Tim Stiles Vanessa, Grant Nick Mawby , ISBN 978-­‐1-­‐904610-­‐00-­‐7 - EMA/INS/GCP/532137/2010 28 February 2012 Reflection paper for laboratories that perform the analysis or evaluation of clinical trial samples   In general, the following consensus recommendations were generated in the process of creation of this FLUCOP SOP:   - Determine the lower and upper limit of quantification of the assay with the selected ELISpot reader. It is important to determine the analytical range in which samples can be measured without dilution with an acceptable level of imprecision. Samples should be further diluted when responses are observed beyond that range. - Reporting of non-numerical values (i.e. in the case of a non-measurable sample) should be properly distinguishable from numerical results. |
| --- |

ELISpot procedure Day 1 - ELISpot plate coating and blocking

Depending on the choice of whether pre-coated plates or plates that still need to be coated will be used in the lab, the plates will be blocked according to the manufacturer’s instructions or the following instructions.

- Label each ELISpot plate according to the plate layout, also add plate number and initials.
- Wash the plates 4 times with 200 µL PBS 1x.
- Block the plates by adding 200 µL cRPMI (containing at least 10 % of the same FBS used for the cell suspensions) to each well.
- Seal the plate(s) with micropore tape and incubate for at least 2 hours (max. 4 hours) at 37°C 5%CO_2_.

## ELISpot procedure Day 1 - Thawing of PBMC

- Prepare one 15 mL conical tube per sample that will be analyzed in the laminar flow bench: label and fill with 7 mL cRPMI.
- Immediately transfer the cryovials from a liquid nitrogen-filled container to the water bath (37°C ± 1°C). Thaw the cryovials in small batches at a time.
- Transfer the PBMC to a 15 mL conical tube filled with 7 ml cRPMI.
- Centrifuge at 350 g for 7 minutes at room temperature (RT).
- Remove the supernatant from the 15 mL conical tubes.
- Resuspend cells in 5 mL benzonase solution (cRPMI with benzonase at a concentration of 25 units/mL).
- Incubate at (37°C ± 1°C) for 10 minutes in a water bath.
- Centrifuge at 350 g for 7 minutes at RT.
- Remove the supernatant.
- Resuspend the cells in 2 ml cRPMI and let them rest in a humidified 5% CO_2_ incubator with the cap slightly loose for 3 hours (resting of cells).
- Centrifuge at 350 g for 7 minutes at RT.
- Remove the supernatant.
- Resuspend cells in 2 mL culture medium and count the cells and determine cell viability.

| Recommendation:  Counting of the cells can be executed using manual or automated methods. However, it is important to validate the selected method and to evaluate the proficiency of the executing operators. |
| --- |

- Centrifuge the cells at 350 g for 7 minutes at RT.
- Remove the supernatant from the washed cells.
- Resuspend the cells in cRPMI at a final cell concentration of 4x10^6^ cells/mL.

Note: take into account the volume remaining with the cell pellet when resuspending the cells

## ELISpot procedure Day 1 - Preparation of stimuli

Recommendation:

Stimulating agents (peptides, proteins, virus-like particles (VLPs), …) should be pre-tested to evaluate the optimal stimulation concentration and potential cell cytotoxicity.

## ELISpot procedure Day 1 - Cell stimulation

- Discard the blocking solution
- Add 100 µL/well of stimulation or mock solution. Analyze each test condition (stimulation or mock) in triplicate: three wells/condition/sample.

Note: It is recommended to physically separate wells containing the superantigen SEB from regular wells to avoid cross-contamination.

- Plate 200.000 cells/well by adding 50 µL of cell suspension/well.
- Seal the closed plates with micropore tape to avoid evaporation.
- Gently shake the ELISpot plate(s) horizontally to divide the cells equally over the wells. Check the water level of the incubator before the start of incubation and make sure the incubator is well leveled to avoid grouping of cells at the border of the wells.
- Incubate overnight at 37°C and 5% CO_2_. Do not move the plates during incubation, this can negatively affect spot development.

| Recommendation:  FLUCOP observed no remarkable differences between protocols incubating cells between 18 and 24 hours. Therefore FLUCOP recommends incubating cells not less than 18 hours and not longer than 24 hours. |
| --- |

## ELISpot procedure Day 2

- Dilute the selected detection antibody at pre-determined (titrated) optimal concentration in filtered PBS 1x containing 0.5% heat inactivated-FBS. Where needed pre-filter the antibody solution with a 0,22 µm low-adsorption filter to remove aggregates.
- Wash the plates 5 times with 200 µL/well lab-grade water.

| Recommendation:  Plates can be washed using either manual or automated procedures. It is good practice to pre-validate the selected method and/or equipment. |
| --- |

- Add 100 µL of the detection antibody at the optimal concentration (e.g. 1/400) to each well and incubate the plate(s) for 2 hours at RT.

Note: Follow the manufacturer’s instructions for additional detection antibody and streptavidin-enzyme conjugate steps

- Wash the plate 3 times with 200 µL/well water and then 3 times with 200 µL/well PBS 1x
- Add 50 µL of substrate to each well (substrate was removed from refrigerator 30 min in advance and filtered as well).
- Let the plate(s) rest for 10 minutes until clearly visible spots are developed.
- Stop the color development by washing extensively with lab-grade water. Remove the under drain (the soft plastic under the plate) and rinse the back of the membranes. Flick off all excess water.
- Leave the plate to air dry in the dark.
- Inspect and count the spots in the ELISpot Reader.

| Recommendation:  Applied ELISpot plate readers should be properly maintained and controlled on regular bases. Staff operating the ELISPOT readers should be appropriately trained and certified. |
| --- |

- Store plates in the dark at room temperature for no longer than 1 month before spot enumeration.
- Report results after subtraction of the background (unstimulated) response as Spot Forming Units (SFU) per million of PBMC.

Recommendation:

Use well-defined flagging codes to indicate if errors occurred or if certain predefined quality criteria have not been met.
