# Supplementary material for: Harmonization and qualification of an IFN-γ Enzyme-Linked ImmunoSpot assay (ELISPOT) to measure influenza-specific cell-mediated immunity within the FLUCOP consortium
Source: Front Immunol. 2022 Sep 8;13:984642. doi: 10.3389/fimmu.2022.984642 (PMC9493492; doi:10.3389/fimmu.2022.984642)
Supplement: Supplementary file 2 [file DataSheet_2.docx]

#
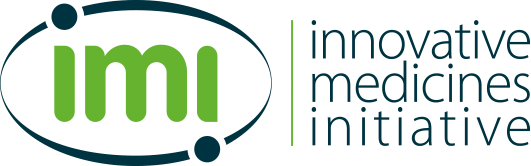


**
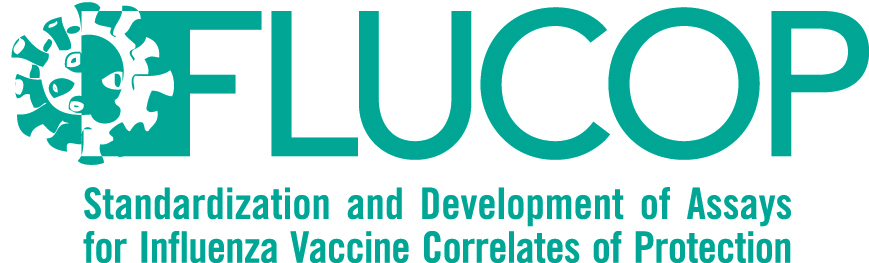
**

**Standard Operating Procedure**

**for isolation, cryopreservation and shipment of PBMC**

Table of Contents

[1](#_Toc475484006)

[INTRODUCTION 3](#_Toc475484007)

[REAGENTS 3](#_Toc475484008)

[MATERIALS and EQUIPMENT 4](#_Toc475484009)

[TRAINING AND PROFICIENCY 4](#_Toc475484010)

[PROCEDURES 4](#_Toc475484011)

[Blood collection 4](#_Toc475484012)

[Preparative work 5](#_Toc475484013)

[PBMC isolation 5](#_Toc475484014)

[Classical separation tubes with LymphoprepTM 5](#_Toc475484015)

[ACCUSPIN^TM^ tubes with Histopaque®-1077 7](#_Toc475484016)

[Cell Preparation Tubes (CPTTM) 9](#_Toc475484017)

[Cryopreservation and Shipment 10](#_Toc475484018)

[General considerations. 10](#_Toc475484019)

[Procedure 11](#_Toc475484020)

[RECOMMENDATIONS 12](#_Toc475484021)

[Recommendation 1 – Blood collection tubes 12](#_Toc475484022)

[Recommendation 2 – Separation tube and media 13](#_Toc475484023)

[Recommendation 3 – Medium to dilute the blood and wash the cells 14](#_Toc475484024)

[Recommendation 4 – Freeze medium 15](#_Toc475484025)

[REFERENCES 16](#_Toc475484026)

# INTRODUCTION

The overall objective of work Package (WP) 2 within the FLUCOP project was to advance the understanding and application of cell-mediated immunity (CMI) assays as tools for evaluating the immunogenicity of influenza vaccines. One of the tasks was to create a standardized protocol for the preparation and cryopreservation of Peripheral Blood Mononuclear Cells (PBMC).

The collaboration of three academic partners and one industrial partner resulted in the conduct of a multicentre experiment. The design of the experiment consisted of 12 test protocols that had to be executed by 4 centers. The protocols included a set of critical parameters that had been selected out of the Standard Operation Procedures for the isolation, cryopreservation and shipment of PBMC collected from WP2 partners. The following critical parameters were identified: (1) blood collection tubes and anticoagulants, (2) culture medium used to dilute blood and to use in wash steps, (3) separation tubes and media and (4) freeze medium. The test protocols were designed in such a manner that they allowed investigating the impact of each individual parameter on the quality of cell preparation and preservation. Participating laboratories have processed blood samples according to these proposed test protocols. In parallel, they have also used their in-house protocol. The integrity and functional qualities of the isolated cells were analysed using interferon-γ ELISpot and ICS (intracellular cytokine staining) technologies. Statistical analyses were applied to investigate the impact of the critical parameters on a set of predefined quality criteria, namely: at least 80% viability, a yield of at least 50% and a background response not exceeding 0,1% of parent (ICS) or 100 SFU/10^e^6 cells (ELISpot).

The main conclusion of this experiment is that the impact of the different test protocols with varying critical parameters is minimal as long as some general guidelines are followed and general good practices are applied. Based on these observations, we present hereafter **a standard operating procedure (SOP) with some recommendations**. In this SOP, the procedure for PBMC isolation, cryopreservation and shipment is described based on a literature review, general good practices, common accepted procedural steps and includes four recommendations related to the selected critical parameters.

# REAGENTS

Reagents are critical in the PBMC isolation and cryopreservation process. The blood samples need to be collected and prepared in a sterile manner to enable proper assessment of immune activation status. The tissue culture media need to contain sufficient nutrients to safeguard the structural and functional integrity of the cells and allow them to respond adequately upon activation. The following are considered to be critical reagents: the cell separation medium (density gradient medium), the cryoprotectant (DMSO), any protein matrices used (i.e. heat-inactivated foetal bovine serum (HI-FBS)), any buffers and media used in the process.

It is good practice to trace the lot numbers of the used reagents, their expiration dates and certificates of analysis. Foetal bovine serum should be validated before use to check whether it provides sufficient cell-culture support without generating undesirable background noise.

# MATERIALS and EQUIPMENT

Using good quality lab materials will ensure a good quality of the processed sample. Plastic disposables need to be of lab-grade quality and sterile (packed individually). Changing suppliers or brand should trigger a change control process to avoid accidental misfortune and subsequent sample loss. Equipment is crucial for the success of the process. These should be well designed and fully qualified (based on the method suitability and established standard) before taking into production. Proper control, maintenance and requalification after technical intervention should assure good performance throughout their lifecycle.

# TRAINING AND PROFICIENCY

In today’s highly automated lab, the process of PBMC isolation and cryopreservation remains one of the few fully manual procedures that requires skilled and experienced personnel. Proper execution of the procedures in a timely manner requires a high level of experience. Therefore, it is good practice first to train the technician and obtain proof of their proficiency before engaging the person in PBMC isolation for clinical trials purposes.

# PROCEDURES

## Blood collection

Collect blood by venepuncture using sterile blood collection tubes containing an appropriate anticoagulant such as (lithium) heparin or EDTA. Alternatively, one can opt for blood collection tubes pre-filled with a cell separation medium such as the “Cell Preparation Tubes (CPT^TM^)”.

*Consult* ***Recommendation 1*** *for more information on the choice of blood collection tubes.*

After venepuncture, tubes with anticoagulant should be inverted gently to enable proper mixing of blood and anticoagulant. Ensure that the whole anticoagulated blood samples are transported at room temperature (RT; 18 to 25°C). If ambient temperatures may fluctuate significantly, styrofoam boxes can be used to ensure room temperature during transfer. The quality of the finally obtained PBMC will inversely correlate with the time between the moment of venepuncture and the start of cryopreservation. Ideally, samples can be processed and cryopreserved within 6h after venepuncture. Samples not processed within 24h will generate poor results^1^.

## Preparative work

Prepare a sufficient number of separation tubes with the appropriate separation medium.

- When density gradient medium (Lymphoprep^TM^, Histopaque^®^-1077, Ficoll-Paque^TM^ or equivalent) is used, add 3 mL medium to 15 mL conical tubes and store them in the fridge (2 to 8°C).
- ACCUSPIN^TM^ tubes with Histopaque^®^-1077: Pre-equipped tubes are ready for use; otherwise, fill the tubes with cell separation media following the manufacturer’s instructions.
- Cell Preparation Tubes (CPT^TM^): ready for use.

Prepare a sufficient volume of freeze medium and filter immediately after preparation using a 0,22 µm filter.

*For more information on the choice of freeze medium, read* ***Recommendation 4****.*

Ensure that sufficient controlled-rate freezer containers (e.g. Nalgene^®^ Mr. Frosty, Cool Cell^®^ Freezing Container or equivalent) are available. Alternatively, a qualified controlled-rate freezer can be used. Both methods should ensure minimal cellular dehydration and degradation.

It is generally good practice to register the timings of all procedural steps, and identify the operator and the lot numbers of critical reagents and equipment used. This may facilitate investigations whenever procedural problems arise.

## PBMC isolation

Depending on the choice of separation tubes and media, execute the following steps in a laminar flow cabinet.

*For more information on the choice of method of isolation, read* ***Recommendation 2****.*

### **Classical separation tubes with density gradient medium**

- Dilute the blood sample no more than 1 in 3 volumes (and not less than 1 in 2 volumes) with an appropriate buffer in a sterile recipient.

*Recommendations on the medium to dilute the blood with and as reagent used in the wash steps can be found in* ***Recommendation 3****.*

- Mix the diluted blood by gentle pipetting. Do not press the pipet tip into the bottom of the tube to avoid shear stress.
-
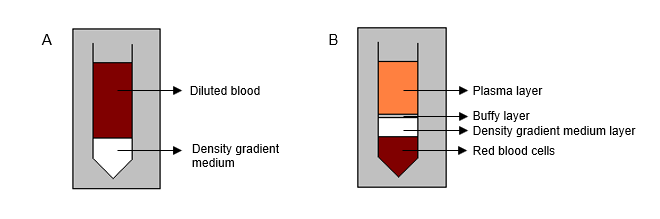
Carefully and slowly layer 10 mL diluted blood on top of 3 mL density gradient medium such that the layer of blood and the layer of medium do not mix (see Figure 1 (A)). Try to minimise the time the blood remains on the cell separation media as a prolonged contact may induce cytotoxicity or activate the cells.

Figure 1. (A) Two layers should be obtained, the diluted blood is on top of density gradient medium (Lymphoprep^TM^) (B) After centrifugation of the filled separation tube, four layers can be distinguished.

- Hold the tubes in an upright position and gently place them in the centrifuge bucket(s).
- Centrifuge at 900 g for 20 minutes at RT with the brake OFF.
- Gently remove the tubes from the centrifuge.
- Collect the cells at the interphase by carefully inserting a 2 or 5 mL pipette at the PBMC layer (buffy layer) located between the diluted plasma and the density gradient medium (see Figure 1 (B)). Take care not to aspirate any more separation medium solution and/or plasma-diluent solution than strictly needed. If applicable, the diluted plasma can be collected for serological applications.
- Collect the cells in 1 or more (depending on the initial number of separation tubes) 50 mL conical tubes per subject.
- Suspend the collected cells in an appropriate wash medium at least 1 in 5 volumes and mix gently. (*see* ***Recommendation 3****)*.
- Centrifuge the diluted cell suspension at 450 g for 10 minutes at RT.
- Aspirate the supernatant without disturbing the cell pellet.
- Re-suspend each pellet with 10 mL of the appropriate wash medium in a 15 mL conical centrifuge tube.
- Centrifuge the diluted cell suspension at 450 g for 10 minutes at RT.
- Aspirate the supernatant without disturbing the cell pellet.
- Re-suspend each pellet with 10 mL of the appropriate wash medium in a 15 mL conical centrifuge tube.
- Count the cells using a validated cell counting method that can discriminate white blood cells from red blood cells. A minor fraction of red blood cells may contaminate the PBMC and it can be difficult to distinguish small lymphocytes from red blood cells only on size.

### **ACCUSPIN^TM^ tubes with Histopaque®-1077**

- Centrifuge the pre-filled ACCUSPIN^TM^ tubes at 500 g for 1 minute at RT to ensure all Histopaque^®^-1077 is in the chamber below the frit. Bring the separation tubes to RT before use.
- Dilute blood sample not more than 1 in 3 volumes (and not less than 1 in 2 volumes) of an appropriate buffer in a sterile recipient. *Recommendations on medium to dilute the blood and as reagent used for washing can be found in* ***Recommendation 3****.*
- Mix the diluted blood by gentle pipetting. Do not press the pipet tip into the bottom of the tube to avoid shear stress.
- Use a sterile pipet to transfer the diluted blood into the ACCUSPIN^TM^ tubes, respecting the maximum allowed volumes: maximum 30 mL diluted blood in a 50 mL ACCUSPIN^TM^ tube (see Figure 2 (Left)).


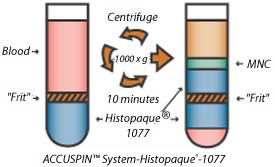


Figure 2. (Left) The diluted blood is separated from the Histopaque®-1077 by a frit, a porous high-density polyethylene barrier. (Right) On centrifugation, the erythrocytes and granulocytes descend through the frit to pellet below the Histopaque®-1077.

- Hold the tubes in an upright position and gently position these in the centrifuge bucket(s).
- Centrifuge at 900 g for 15 minutes at RT with the brake OFF.
- Gently remove the tubes from the centrifuge.
- Collect the cells in 1 or more (depending on the initial number of separation tubes) 50 mL conical tubes per subject.
- Suspend the collected cells in an appropriate wash medium at least 1 in 5 volumes (*see* ***Recommendation 3***) and mix gently.
- Centrifuge the diluted cell suspension at 450 g for 10 minutes at RT.
- Aspirate the supernatant without disturbing the cell pellet.
- Re-suspend each pellet with 10 mL of the appropriate wash medium in a 15 mL conical centrifuge tube.
- Centrifuge the diluted cell suspension at 450 g for 10 minutes at RT.
- Aspirate the supernatant without disturbing the cell pellet.
- Re-suspend each pellet with 10 mL of the appropriate wash medium in a 15 mL conical centrifuge tube.
- Count the cells using a validated cell counting method that can discriminate white blood cells from red blood cells. A minor fraction of red blood cells may contaminate the PBMC and it can be difficult to distinguish small lymphocytes from red blood cells only on size.

### **Cell Preparation Tubes (CPT^TM^)**

- Before centrifugation, remix the blood by gently inverting the tubes 8 to 10 times.
- Centrifuge at 1800 g for 30 minutes at RT with the brake OFF.
- Remove the tubes from the centrifuge.
- These tubes have been developed to enable fast isolation and the layers above the gel plug can be poured directly out of the tube and processed immediately with the hereunder described procedure).


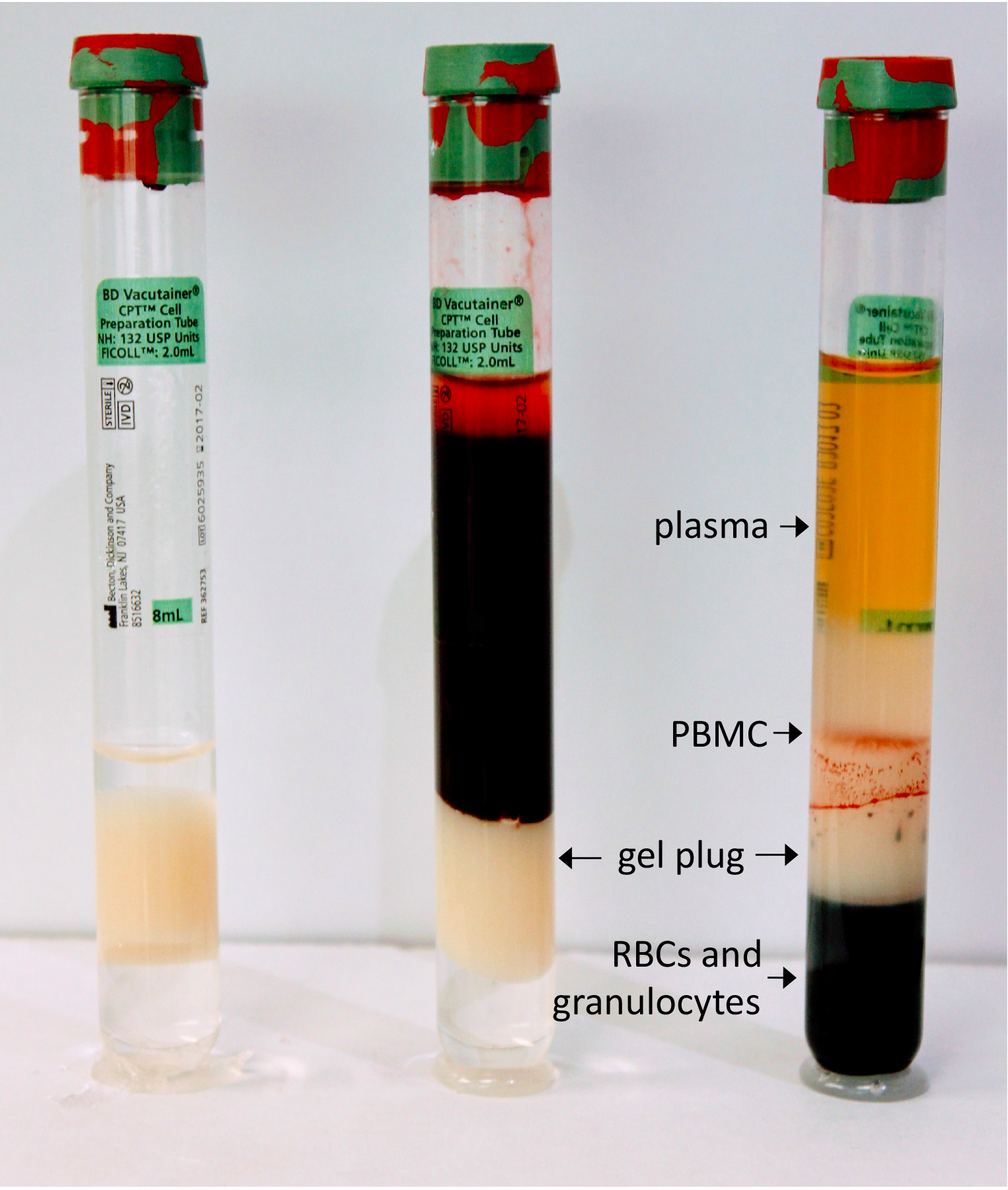


Figure 3. The Cell Preparation Tubes (CPT^TM^, left image) are vacutainer blood draw tubes that contain Ficoll-Hypaque and a gel plug that separates the Ficoll solution from the blood drawn. Blood can be directly collected in the separation tube (middle). Following centrifugation, PBMC are separated from other blood components (right image).

- Collect the cells in 1 or more (depending on the initial number of separation tubes) 50 mL conical tubes per subject.
- Suspend the collected cells in an appropriate medium at least 1 in 5 volumes *(see* ***Recommendation 3****)* and mix gently.
- Centrifuge the diluted cell suspension at 450 g for 10 minutes at RT.
- Aspirate the supernatant without disturbing the cell pellet.
- Re-suspend each pellet with 10 mL of the appropriate wash medium in a 15 mL conical centrifuge tube.
- Centrifuge the diluted cell suspension at 450 g for 10 minutes at RT.
- Aspirate the supernatant without disturbing the cell pellet.
- Re-suspend each pellet with 10 mL of the appropriate wash medium in a 15 mL conical centrifuge tube.
- Count the cells using a validated cell counting method that can discriminate white blood cells from red blood cells. A minor fraction of red blood cells may contaminate the PBMC and it can be difficult to distinguish small lymphocytes from red blood cells only on size.

# Cryopreservation and Shipment

## General considerations.

Storage at very low temperatures is known to provide an indefinite longevity to cells. Laboratories have stored PBMC in Ultra-Low Temperature (ULT) freezers (<-70°C), -150°C freezers and in liquid nitrogen. However, temperatures below the glass transition point (Tg) of polyols in water solutions, around −136 °C (137 K; −213 °F), are accepted as the range where biological activity slows down substantially, and −196 °C (77 K; −321 °F), the boiling point of liquid nitrogen, is the preferred temperature for storing valuable specimens. It is feasible to keep samples at temperatures of -70°C for a limited period of time, enabling transfer from one site to another using dry ice instead of ‘dry-shippers’ pre-filled with liquid nitrogen. However, when kept at -70°C for 3 to 12 weeks prior to shipment, cellular viability and function of PBMC decrease progressively^2^. Unless the thawing process was initiated, shifting PBMC that are kept in liquid nitrogen (-196°C) to temperatures above -136°C should be avoided at all costs^[[1]](#footnote-1)^ as this might re-activate enzymatic processes. Liquid nitrogen storage and shipment grant a quality of cryopreserved PBMC that is not matched by either liquid nitrogen storage combined with dry ice shipment or -70°C storage combined with dry ice shipment. This results in a significant decrease in viability and viable recovery^3^. It is good practice to handle cryopreserved PBMC samples in a liquid nitrogen benchtop container wearing proper safety equipment (protection glasses, gloves,...).

## Procedure

- Centrifuge the final PBMC suspension (with defined cell count) at 350 g for 10 min at RT.
- Discard the supernatant.
- Suspend carefully the cells in freeze medium (pre-chilled at 2-8°C). Apply gentle pipetting and make sure no clumps of cells are visible.

  A rich or poor medium can be selected, depending on the percentage of FBS and/or number and quantities of the supplements added. *For more information on the choice of freeze medium, read* ***Recommendation 4****.* It is generally good practice not to cryopreserve PBMC at concentrations higher than 20 million cells/mL.
- Aliquot the suspended cells in the cryovials (e.g. Nunc 1.8 mL cryotubes) and transfer them immediately to a controlled-rate freeze container (e.g. Nalgene^®^ Mr. Frosty, Cool Cell^®^ Freezing Container, …) and place the container in the Ultra-Low Temperature (ULT) freezer at < -70°C during at least 24h. Alternatively, a qualified controlled rate freezer can be used.
- Transfer the vials to a liquid nitrogen container or -150°C mechanical freezer. Try to minimise the duration of transport and any fluctuations in temperature during transport.

# RECOMMENDATIONS

## Recommendation 1 – Blood collection tubes

FLUCOP WP2 investigated this parameter with the following variables: CPT^TM^, commercial blood collection tubes coated with heparin, commercial tubes coated with EDTA.

- Lithium heparin coated blood collection tubes (BD – art no 367526)
- EDTA coated blood collection tubes (BD – art no 367525)
- Vacutainer^®^ CPT^TM^ Mononuclear Cell Preparation Tube (Sodium Heparin; Ficoll^TM^ Hypaque^TM^ Solution – BD – art no 362780)

No statistically significant differences in the quality of the PBMC were observed between these types of separation tubes and media. Trends observed were:

- (ICS analysis) Viability upon thawing was in general higher than 80% (even higher than 97%).
- (ICS analysis) The quality criterion of a recovery of at least 50% was not always met. Especially the use of CPT tubes resulted in a lower recovery.
- (ICS analysis) The background response did not exceed 0,1% of parent and therefore the third quality criterion was met.
- (ICS analysis) The pre- and post-culture viability were the highest when the in-house protocols were applied.
- (ELISpot analysis) The viability upon thawing and after overnight resting did not always exceed 80%.
- (ELISpot analysis) The background response did not exceed 100 SFU/10e6 cells.
- (ELISpot analysis) The average antigen response measured with samples of the test protocols, except protocols using EDTA tubes, was slightly higher than measured with samples of in-house protocols.

## Recommendation 2 – Separation tube and media

FLUCOP WP2 investigated this parameter with the following variables:

- Vacutainer^®^ CPT^TM^ Mononuclear Cell Preparation Tube (Sodium Heparin; Ficoll^TM^ Hypaque^TM^ Solution – BD – art no 362780)
- ACCUSPIN^TM^ System-Histopaque^®^-1077 tubes (pre-filled) (Sigma Aldrich – art. no A7054)

Principle: Principle: ACCUSPIN^TM^ System-Histopaque^®^-1077 employs centrifuge tubes specially designed with two chambers separated by a porous high-density polyethlyene barrier ("frit"). The lower chamber contains Histopaque^®^-1077 which allows the addition of anticoagulated whole blood without risk of mixing with the separation medium. On centrifugation, erythrocytes and granulocytes descend through the frit to pellet below the Histopaque^®^-1077. The erythrocytes aggregate, increasing their sedimentation rate, resulting in pelleting at the bottom of the ACCUSPIN^TM^ tube. Lymphocytes and other mononuclear cells, i.e., monocytes, remain above the frit at the plasma – Histopaque^®^-1077 interphase. (<http://www.sigmaaldrich.com/life-science/cell-biology/hematology-and-histology/accuspin-1077.html>)

- Standard conical polystyrene tubes filled with Lymphoprep^TM^ (Elitech – art no 1114547)

No statistically significant differences in the qualities of PBMC were observed between these types of separation tubes and media. Trends observed were:

- (ICS analysis) Viability upon thawing was with each condition higher than 80%.
- (ICS analysis) The quality criterion of a recovery of at least 50% was not always met. Especially the use of CPT tubes resulted in a lower recovery.
- (ICS analysis) Background response did not exceed 0,1 % of parent.
- (ICS analysis) Within the CD8 population, PBMC isolated by using the Accuspin tubes gave a slightly lower response upon polyclonal stimulation.
- (ICS analysis) Within the CD4 population, PBMC isolated using the CPT tubes demonstrated a slightly lower response upon CEF stimulation.
- (ELISpot analysis) The viability upon thawing and after overnight resting did not always exceed 80%.
- (ELISpot analysis) Background did not exceed 100 SFU/10e6 cells. The average background response was slightly higher when using CPT tubes.

HANC (HIV/AIDS Network Coordination) recommends the use of either Cell Separation Tubes with Frit Barrier (CSTFB) or manual density gradient cell separation and provides a Standard Operating Procedure with each of these^4^.

## Recommendation 3 – Culture medium used to dilute the blood and to use in wash steps

FLUCOP WP2 investigated this parameter with the following variables:

- Poor medium: NaCl 0.9%
- Rich medium:

10% FBS (Sigma)
89% RPMI-1640 w/ L-Glutamin (Lonza – art no BE12-702F)
1% Penicillin-streptomycin-Fungizone (Lonza – art no 17-745E)
Gentamycin (50 μg/ml) (Thermofisher – art no 15750-060)

No statistically significant differences were observed between these types of media. Trends observed were:

- (ICS analysis) The viability upon thawing was with both conditions higher than de predefined threshold of 80%. The difference in averages measured in samples processed with either rich medium or poor medium was only 0,5 %.
- (ICS analysis) The quality criterion for recovery (at least 50%) was not always met. Poor medium gave a slightly higher recovery.
- (ICS analysis) Background did not exceed 0,1 % of parent. Rich medium gave a slightly higher background response within the CD4 population.
- (ICS analysis) The average response upon polyclonal stimulation within CD8 population was higher when using a poor medium.
- (ELISpot analysis) The viability upon thawing and after overnight resting did not always exceed 80%.
- (ELISpot analysis) The background response did not exceed 100 SFU/10e6 cells.
- (ELISpot analysis) The average response upon antigen stimulation was higher when using poor medium.

HANC (HIV/AIDS Network Coordination) recommends the use of either Hanks’ Balanced Salt Solution (HBSS) without calcium or magnesium or Phosphate-Buffered Saline (PBS) without calcium or magnesium (1x)^4^.

## Recommendation 4 – Freeze medium

Studies^3^ and organizations like HANC (HIV/AIDS Network Coordination^4^) recommend the use of 9 parts of heat-inactivated Foetal Bovine Serum (HI-FBS) completed with 1 part DMSO as cryopreservation solution.

Research has been performed to investigate the impact of lowering the content of FBS in freeze medium. A concentration of 40% or 70 % FBS did not show to have significant impact on either cell viability or functionality (antigen response)^5^.

Other research groups investigate the use of serum-free cryomedia to overcome issues related to FBS-containing media such as lot-to-lot variability and import restrictions of specimens containing FBS. In addition, research on alternatives for DMSO has been performed to eliminate the risk of cell toxicity of the reagent at room temperature^6^. Cryopreservation efficiency of serum-free media^6,7,8^ and fully chemical serum free-media was found to be comparable with FBS-containing media during storage of a few weeks and for several months^7^.

# REFERENCES

1. Bourguignon P, Clement F, Renaud F, Le Bras V, Koutsoukos M, Burny W, et al. Processing of blood samples influences PBMC viability and outcome of cell-mediated immune responses in antiretroviral therapy-naive HIV-1-infected patients. J Immunol Methods 2014; 414:1-10.

2. Weinberg A, Song LY, Wilkening CL, Fenton T, Hural J, Louzao R, et al. Optimization of storage and shipment of cryopreserved peripheral blood mononuclear cells from HIV-infected and uninfected individuals for ELISPOT assays. J Immunol Methods 2010; 363:42-50.

3. Higdon LE, Lee K, Tang Q, Maltzman JS. Virtual Global Transplant Laboratory Standard Operating Procedures for Blood Collection, PBMC Isolation, and Storage. Transplant Direct 2016; 2:e101.

4. COORDINATION HAN. CROSS-NETWORK PBMC Processing Standard Operating procedure. 2014:41.

5. Nazarpour R, Zabihi E, Alijanpour E, Abedian Z, Mehdizadeh H, Rahimi F. Optimization of Human Peripheral Blood Mononuclear Cells (PBMCs) Cryopreservation. Int J Mol Cell Med 2012; 1(2):88-93.

6. Germann A, Schulz J, Kemp-Kamke B, Zimmermann H, von Briesen H. Standardized Serum-Free Cryomedia Maintain Peripheral Blood Mononuclear Cell Viability, Recovery and Antigen-Specific T-Cell Response Compared to Fetal Calf Serum-Based Medium. Biopreserv biobank 2011;9(3):229-236.

7. Schulz J, Germann A, Kemp-Kamke B, Mazotta A, von Briesen H, Zimmerman H. Towards a xeno-free and fully chemically defined crypreservation medium for maintaining viability recovery, and antigen-specific functionality of PBMC during long-term storage. J Immunol Methods 2012;382(1-2):24-31.

8. Bull M, Lee D, Stucky J, Chiu YL, Rubin A, Horton H, McElrath MJ. Defining blood processing parameters for optimal detection of cryopreserved antigen-specific responses for HIV vaccine trials. J Immunol Methods 2007;322(1-2):57-69.

1. [↑](#footnote-ref-1)
