# Supplementary material for: Harmonization and qualification of an IFN-γ Enzyme-Linked ImmunoSpot assay (ELISPOT) to measure influenza-specific cell-mediated immunity within the FLUCOP consortium
Source: Front Immunol. 2022 Sep 8;13:984642. doi: 10.3389/fimmu.2022.984642 (PMC9493492; doi:10.3389/fimmu.2022.984642)
Supplement: Supplementary file 3 [file DataSheet_3.docx]

Supplementary Material





**Supplementary Figure 1.** **Determination of optimal concentration of split A/California (H1N1) virus.** PBMC of 5 different donors were stimulated with increasing concentrations of split A/California (H1N1) virus. Mean IFN-ɣ ELISpot background-subtracted results are shown. Error bars represent standard deviation. The highest mean responses were observed at 1.25 µg/mL split A/California virus.





**Supplementary Figure 2. Determination of the ULOQ.** PBMC from two samples were plated each on one plate in a serial dilution, ranging from 100.000 to 48 PBMC per well. Data from sample 2 is shown. Each condition was repeated 8 times and all cells were stimulated with SEB. Mean SFU per dilution with SD bars are indicated in blue and represented on the left y-axis. The related CV% are shown in black and represented on the right x-axis. The dotted line indicates the acceptance criterion of 40% CV.
